# RAMSMART: a low-invasive system for real-time automated multi-species monitoring of livestock activity in research trials

**DOI:** 10.3389/fvets.2026.1830138

**Published:** 2026-06-22

**Authors:** Harmen P. Doekes, Cees J. Voesenek, Rineke de Jong, Ines Adriaens, Bing Han, Norbert Stockhofe-Zurwieden

**Affiliations:** 1 Wageningen Bioveterinary Research, Wageningen University and Research, Lelystad, Netherlands; 2 Wageningen University and Research Animal Breeding and Genomics, Wageningen, Netherlands; 3 VORtech BV, Delft, Netherlands; 4 Livestock Technology Group, Department of Biosystems, Division of Animal and Human Health Engineering, KU Leuven, Leuven, Belgium

**Keywords:** accelerometry, behavior, early warning system, laboratory animals, precision livestock farming, sensing, welfare monitoring

## Abstract

**Background:**

The continuous and automated monitoring of animal activity with sensors can help to refine and improve the quality of animal experiments. The aim of this study was to develop a system for real-time automated multi-species monitoring of individual animal activity in research trials (“RAMSMART”), to test this system in terms of battery life and data completeness, and to demonstrate its added value.

**Methods:**

We used lightweight commercially available accelerometers with edge computing and uploaded self-written firmware to these accelerometers to calculate the vectorial dynamic body acceleration (VeDBA). The accelerometer is attached, for example, to an animal’s ear tag. It then collects raw acceleration data (at a user-defined sampling frequency), calculates the mean VeDBA across a certain period (based on a user-defined updating interval) and creates a data packet with the VeDBA, which is then broadcasted (at a user-defined broadcasting frequency) through Bluetooth Low Energy to a nearby receiving device, e.g., a laptop. On this receiving device, data are stored in a SQLite database and regularly copied to a shared drive to enable (near) real-time visualization of results from outside animal facilities.

**Results:**

Battery life depended on the sampling frequency, the updating interval and the broadcasting frequency. For example, when the sampling frequency was kept constant at 12.5 Hz, and the broadcasting frequency at 4 Hz, an updating interval of 1 second or 1 minute resulted in a battery life of 15.9 days (± 1.0 days) or 47.7 days (± 1.9 days), respectively. To avoid missing data, a sufficiently high broadcasting frequency was needed and the distance between sensor and receiver should be limited (preferably to < 20 m), or a multiple receiver set-up could be used. We showcased RAMSMART in three infectious disease trials in pigs, sheep and calves, connecting the VeDBA with other clinical symptoms, and demonstrating the added value of this system for early detection of deviations in activity.

**Conclusion:**

RAMSMART provides a low-invasive, affordable, re-usable, generalizable and plug-and-play approach for routine and long-term real-time automated activity monitoring based on acceleration data of (particularly livestock) animals in research trials. The source code and documentation of RAMSMART are openly available^1^.

## 1 Introduction

While animal experimentation is under continuous societal debate, animal models remain the golden standard for various purposes for which there are no adequate alternatives. Beyond rodents and fish, livestock species are commonly used in animal research, for example to study infectious diseases and to evaluate the effectiveness of human and animal vaccines. Annually, more than 400,000 domestic fowl (primarily chickens) and 120,000 farm mammals (such as pigs, cattle, and sheep) are used in the European Union for research purposes (1). Part of these animals are kept in a small-scale indoor setting for a couple of weeks to a couple of months, and in high containment conditions.

From regulatory and ethical considerations, researchers should implement the 3R-framework to replace, reduce and refine animal studies. When no adequate alternatives exist to replace the animal model, the number of animals should be reduced where possible and studies should be refined to minimize animal suffering (2, 3). To reduce the number of animals, it is among others important to maximize the amount of information that is collected per individual animal. To refine animal studies, it is important to monitor animal welfare and intervene timely when needed, for instance by applying humane endpoints (3, 4). This is particularly important given that animal studies involve experimental treatments, such as challenges with infectious diseases, that have a (temporary or lasting) negative effect on animal welfare.

Animal behavior is an important indicator of animal welfare. Animal behavior is a key component in both traditional and recent welfare assessment strategies, such as the five freedoms model (5) and the five domains model (6). This includes the occurrence of favorable behaviors, such as (allo) grooming and play behavior, and unfavorable behaviors, such as damaging behaviors. Sickness behaviors, such as reduced levels of activity, reduced appetite and social withdrawal, are indicators of a negative welfare state induced by a disease (7, 8). While sickness behaviors indicate a welfare impairment, they are also an adaptive coping mechanism that can help the animal to overcome the disease more rapidly, e.g., by redirecting resources from movement to immune response (7, 8). Continuous monitoring of (sickness) behaviors in animal research trials is, therefore, not only valuable in the context of an early warning system, but it also provides deeper insight into how different groups of animals—such as treatment and control groups—respond to an experimentally induced challenge.

Traditionally, the behavior of animals in research trials is monitored through visual observations carried out once or twice daily by researchers or biotechnicians. While such visual observations are informative, they are also prone to inter and intra-observer biases, are interventional, are infrequent and are typically only taken during daytime (9). Therefore, they are suboptimal to capture the full temporal dynamics of an animal’s condition. When behavioral deviations occur, e.g., when the activity levels of an animal drop in response to a disease, the onset and duration of these deviations may be incorrectly inferred, or they may be missed entirely (9, 10).

Previous work in the broader field of Precision Livestock Farming (PLF) has demonstrated that sensor-based monitoring can provide continuous, high-resolution, and objective data on animal behavior (11–13). Many (commercial) systems exist to continuously monitor animal behavior, based on sensor technologies like computer vision, global positioning systems (GPS), ultra-wideband (UWB), radio-frequency identification (RFID), and accelerometry. All these technologies have pros and cons related to invasiveness, hardware installation, scalability, spatial resolution, temporal resolution, and costs. Computer vision is rapidly increasing in popularity, as it allows for completely non-invasive animal monitoring with cameras, and similar approaches may be used across species (14). To date, however, it remains challenging to track individual animals that are phenotypically similar (such as pigs, or chickens) for a long time when they are housed in groups, and—when housed in environments with a large surface area—across many field of views without using additional markers (14, 15). Moreover, computer vision approaches typically require substantial data storage and computation time. Hence, other approaches are still commonly used. Body-worn sensors, or “wearables,” are common low-invasive alternatives. Wearable GPS sensors are primarily used to quantify land usage and behavior of extensive grazing livestock (16), perform less well indoors, and have a relatively poor spatial resolution with horizontal errors of a couple of meters (17). Wearable UWB sensors can be combined with beacons placed at the edges of the animal pen to provide informative individual-level positioning data with positioning errors of 0.2 to 0.3 m (18). However, with a weight of ~ 25 grams, these sensors are unsuitable for smaller animals, such as young chickens (19, 20). In addition, the installation and calibration of UWB systems is rather laborious and, for small enclosures in which the beacons cannot be placed outside of the enclosure, tracking animals of the edges of the pen can be challenging. As an alternative, RFID systems can use very lightweight wearable tags, and can provide information on when an animal was present at strategically located antenna throughout the pen (20). However, to obtain high spatial resolution for overall monitoring through time and space, many antennae may be needed, and the installation becomes more complex and costly (20). Accelerometers, which are not based on spatial monitoring but rather measure acceleration in three axes, have been widely applied in commercial livestock contexts to identify movement and resting behavior, and—with lower accuracies—feeding and drinking behavior (13, 21, 22). While accelerometers can be lightweight and applied to various livestock species, available commercial systems are typically designed for a single species and require licenses for automated data processing. All these PLF approaches, combined with automated data processing pipelines, have been shown to facilitate near-real-time early warning systems. However, the translation and implementation of these methods to the specific context of experimental (livestock) research trials have remained limited so far.

The research setting puts requirements on an activity monitoring system, which partly differ from those under the commercial setting. Given that research facilities work with various species and housing systems, the ideal monitoring system should be generalizable across species and pen characteristics, with minimal impact on the animals. In addition, experiments typically last a few weeks to a couple of months, which puts requirements on battery life (to limit repeated handling) and ease of installation. Under high biosafety conditions, as in the case of infectious disease studies, the animal room is completely emptied, and both the room and hardware are disinfected after each study. Thus, hardware should be water and disinfectant proof (at least IP65 rated) and easy to install. Moreover, in case of positioning-based monitoring systems, good spatial resolution is needed. This includes accurate positioning along the edges of the pen, which is challenging for, for example, UWB-systems. Aside from a high spatial resolution, also a high temporal resolution, with (near) real-time and automated data processing, is needed for an early warning system. This implies that (near) real-time analysis should be computationally feasible and requires that sensors can transfer data reliably in real time. Last, for scalability and to make sensor-based behavioral monitoring a common method in experimental trials, the system should be affordable and re-usable across animal studies.

Previous work has shown that the use of accelerometers, with the vectorial dynamic body acceleration (VeDBA) as an activity measure, is a promising approach that meets most of the abovementioned requirements (9). Namely, accelerometers are lightweight and can be used across species, they do not require complex hardware set-ups, can provide high temporal resolution data while maintaining good battery life and provide informative data that can be related to an animal’s behavior and health (9, 13). The VeDBA has been shown to be a robust proxy for overall activity and energy expenditure across species, though this has been particularly used in the wildlife context (23, 24) and to a lesser extent in livestock species (25, 26). In addition, an open-source software system for continuous monitoring of the VeDBA with real-time data transfer is, to the best of our knowledge, not yet available.

The aim of this study was to develop a system for Real-time Automated Multi-Species Monitoring of livestock Activity in Research Trials (RAMSMART). To set up this system, we employed a commercially available accelerometer with edge computing. We built novel and customized firmware that calculates and broadcasts the VeDBA per animal to a nearby receiving device through Bluetooth Low Energy, and uploaded this firmware to the accelerometer. We also incorporated automated visualizations, which can be readily interpreted by researchers and biotechnicians during the research trials. We describe the system architecture and investigate how battery life and data completeness may be affected by three customizable parameters: the sampling frequency (i.e., how often raw acceleration data are sampled), the updating interval (i.e., how often new data packets are created with the latest VeDBA), and the broadcasting frequency (i.e., how often the sensor sends out a data packet per second). In addition, to demonstrate its added benefit to traditional human observations, and to illustrate its multi-species applicability, we showcase the use of RAMSMART in three infectious disease studies in pigs, sheep and calves. In these trials, we hypothesized that RAMSMART would help to better quantify the onset, severity and duration of behavioral deviations induced by the disease challenges, as compared to the (bi-) daily visual observations by bio technicians.

## 2 Materials and methods

### 2.1 Animal care and ethical approval

The infectious disease trials in which RAMSMART was showcased with 16 pigs, 24 sheep and 24 calves (as described in more detail in section 2.6) were performed under legislation of the Dutch Central Authority for Scientific procedures on Animals (CCD licenses no. AVD40100202316677, AVD40100202317648) and approved by the Animal Welfare Body of Wageningen Research before the experiments took place (experiment number 2025_2022. D-0027.005 for the pig trial, 2023. D-0025.002 for the calf trial and 2023. D-0025.001 for the sheep trial). Animal care and the (ethical) assessment were carried out according to the Dutch regulations as stipulated in the “Wet op de dierproeven” and in the “Dierproevenbesluit.” The studies were performed in animal biosafety level 3 laboratories at Wageningen Bioveterinary Research, Lelystad, Netherlands.

### 2.2 Sensor specifications

The sensor we used is the Movesense HR2 (Movesense Ltd., Vantaa, Finland). The accelerometer (±16 g) has a flexible sampling frequency, ranging from 12.5 Hz to 833 Hz. The Movesense HR2 is equipped with default firmware, and self-written firmware can be uploaded to the device for customization using the open development environment (see www.movesense.com/docs). The sensor exploits edge computing, allowing to process the sampled data on the sensor before broadcasting it through Bluetooth Low Energy (BLE 0.4 GHz) to a nearby receiving device (e.g., a laptop). The sensor has a diameter of 36.6 mm, is 10.6 mm thick, weighs 10 grams and is water resistant to 30 meters. It costs ~100 euros per sensor and uses a CR2025 coin cell battery, which costs ~0.60 euros per battery (prices January, 2026).

In total, we used 24 sensors for the purpose of this study. Eight sensors were used for testing, 16 sensors were (re-)used in the pig trial and all 24 sensors were (re-)used in the sheep and calf trials.

### 2.3 Description of RAMSMART

The source code and corresponding documentation for RAMSMART are openly available^2^. In short, the sensor samples acceleration data at a user-defined sampling frequency and the VeDBA is continuously calculated from these data. The VeDBA is averaged over a certain period (based on a user-defined updating interval), placed in a data packet and then broadcasted via BLE. The data packet is broadcasted with a user-defined broadcasting frequency, until the next packet is ready, after which the sensor will start broadcasting the new packet.

The activity measure that is used is the VeDBA. From the raw acceleration data, the VeDBA is continuously computed as (23):


$$\text{VeDB}A_{i}=\sqrt{{\left(x_{i}-\bar{x_{i}}\right)}^{2}+{\left(y_{i}-\bar{y_{i}}\right)}^{2}+{\left(z_{i}-\bar{z_{i}}\right)}^{2}}$$

where $x_{i}$ , $y_{i}$ and $z_{i}$ are the raw accelerations at time i, and ( $\bar{x_{i}}$ ), ( $\bar{y_{i}}$ ) and ( $\bar{z_{i}}$ ) are the rolling averages of the acceleration signals for the three axes (in *g*). The rolling average is subtracted from the raw accelerations to remove the static (gravitational) component and retain the dynamic component (23).

**Figure 1:**
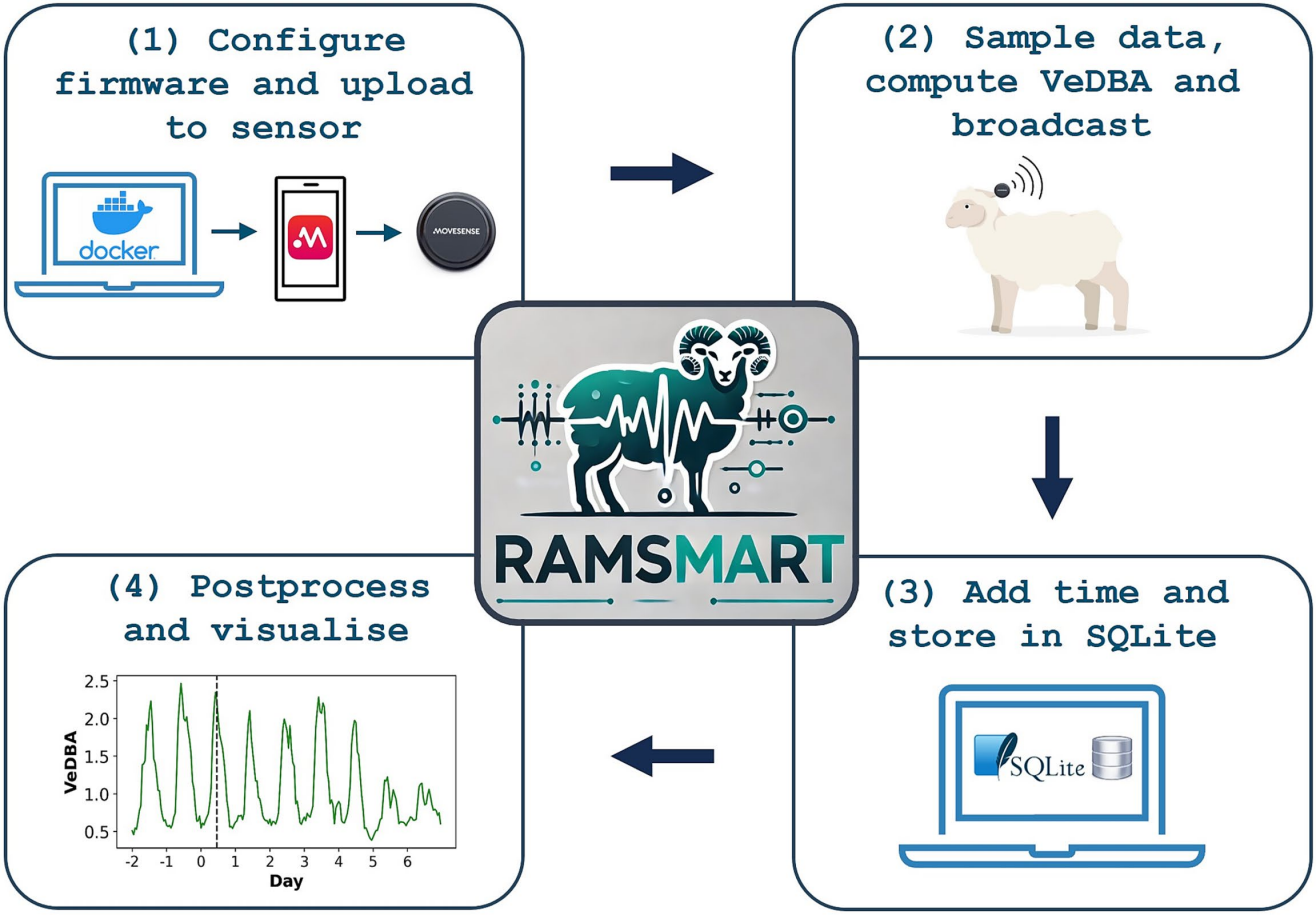
Overview of the four steps of RAMSMART.

The use of RAMSMART can be divided in four steps (Figure 1). In step one, firmware is configured by the user and subsequently build and uploaded to the sensor. In the configuration, five parameters can be customized: the sampling frequency, the interval for the rolling average correction in computation of VeDBA, the updating interval, the broadcasting frequency, and the interval between battery level checks. The sampling frequency (in Hz) is the frequency at which the sensor samples acceleration data, for example 26 Hz. The default interval for the rolling average correction in the computation of the VeDBA is set to 2 s, similar to previous work in sheep (9, 25), but it can be changed if desired, e.g., for other species. The updating interval determines the interval in seconds between updating data packets. For example, when a sampling frequency of 26 Hz is used in combination with an updating interval of 10 s, the data packet is updated every time when the sensor has collected 260 data points, and the broadcasted packet contains the VeDBA averaged over the previous 10 s. The broadcasting frequency is the frequency (in Hz) with which the sensor broadcasts data packets. This value can be changed if data reception is otherwise unreliable, e.g., if many sensors are used with a single receiving device. The interval between battery level checks is set to a default of once every 10 min, but can be changed as well. Once the configuration parameters are set, the firmware can be built, using the firmware-folder from the RAMSMART-repository and Docker Desktop for running the development container. The firmware can be built in Visual Studio Code with the Dev Containers extension, or manually on the command prompt, which takes less than 30 s. A DFU package (represented as a zipped folder) will be created, which should be transferred to a device (e.g., smartphone) on which the Movesense app is installed. In the Movesense app, the firmware can be uploaded to the sensor through the phone’s Bluetooth connection. This is possible on both Android and iOS (our experience is that the iOS version is more straightforward). Uploading the firmware to the sensor on iOS takes ~10 s per sensor.

In step two, the sensor is attached to the animal and data are collected. The sensor is attached to the animal. The preferred attachment strategy may depend on the species and the activity of interest. We prefer to attach the sensor to animals’ ear tags (with duct tape), as a simple and effective approach for species that already wear ear tags for identification purposes. It is important to realize that—in this case—the accelerometer will measure all head-based movement. In chickens, we have previously used customized “backpacks” [similar to those used by (27)] to attach an accelerometer, which was effective but they had to be adjusted when animals grew and sometimes they got loose. In sheep and calves, we previously explored also attaching an accelerometer to the back (with a mating harness in sheep), to a neck collar (in cattle) or to the leg with an elastic band (in cattle). We observed substantial differences in absolute VeDBA levels across ear, back and leg, but general activity trends over time were similar (28). Therefore, for overall monitoring of activity patterns over time—and detecting deviations in activity patterns—we believe that various attachment positions could be used, and the ear tag is least invasive if the animal already wears an ear tag and it is a practical solution given the ease of use and the fact that we have lost none of those ear-worn sensors. If the firmware was successfully built and uploaded in step 1, the sensor will collect data and broadcast the VeDBA. Broadcasting occurs via a Bluetooth advertisement protocol. This protocol is normally used to initiate a connection between the sending BLE device and the receiving device, but also allows for a small amount of data to be encoded in the advertising packet. Since RAMSMART does not require the sensor to receive any input, and only requires a very limited amount of data (13 bytes) to be broadcasted, first initiating a connection is not needed, thereby saving complexity and battery life. Each data packet consists of a unique packet identifier, the number of samples used for calculating the VeDBA, the VeDBA itself and the battery percentage. The battery percentage is an estimate based on a voltage measurement under specific load, which is not highly accurate, but gives an indication of the remaining battery life.

In step three, the broadcasted data should be retrieved by a nearby device, e.g., a laptop. Thus, a laptop (with Bluetooth or Bluetooth adapter) should be placed near the animal pen. The receiving laptop should have Python installed, and the ‘receiver’ component of RAMSMART should be installed. When running the receiver, it listens for all the broadcasted packets in the environment, filters the packets coming from Move sense sensors, adds a sensor identifier (the Bluetooth address of the sensor) as well as a timestamp of receiving the package, and then stores the data in a SQLite database. In a configuration file (.yaml), the user can specify the name of the database, as well as the Bluetooth addresses from which data should be received. In this way, it is possible to have multiple receiving devices next to each other, with each device receiving data from different sensors. Note that the sensor broadcasts the same data packet many imes (depending on the user-defined broadcasting frequency). The unique data packet identifier, which is only updated when a new packet is created (following the updating interval), is used to prevent that the same data is stored multiple times. To enable remote access from outside the animal room, one could for example save (or regularly copy) the database to a shared environment.

Last, in the fourth step, the results are extracted from the SQLite database in real-time, postprocessed, and visualized. For this purpose, we developed a Python script (which can be compiled into an executable) along with two simple input files. In the first input file, the user specifies the start date, treatment date, and end date of the experiment, as well as the desired rolling interval for smoothing the activity patterns (e.g., 3 h). In the second input file, the user specifies which Bluetooth addresses are linked to which animals and, if applicable, to which groups (e.g., to indicate which animals belong to control and treatment groups). Also, animal-specific endpoints can be added. When running the script, various figures are created. For each animal, the smoothed VeDBA pattern across the experiment is plotted, including reference values for the animal’s “normal activity” pattern based on the minimum and maximum VeDBA of the same timepoint for the days before the treatment. In addition, the battery life and data completeness over time are visualized. Moreover, a figure at group level is created to compare the mean (and standard deviation) of the VeDBA across groups over time.

### 2.4 Evaluation of battery life

To evaluate the effect of different settings on battery life, we tested various sampling frequencies, updating intervals and broadcasting frequencies. In total, we used eight unique sensors for testing (before usage in the animal trials). For every combination of settings, we used three randomly selected sensors as replicates.

In a first test, we kept a constant broadcasting frequency (of 4 Hz) and a constant sampling frequency (of 12.5 Hz) and varied the interval of updating the data packet from approximately once every 1 s, to once every 2 s, once every 10 s, and once every minute. Note that these were approximate intervals, because (1) the interval was determined in number of samples, which was the sampling frequency multiplied with the user-defined interval in seconds, and the sampling frequency may have varied somewhat across sensors, and (2) we configured the sensors to sample at 13 Hz, while in reality the sampling frequency was closer to 12.5 Hz (thus, an updating interval of 1 s may actually be closer to 1.04 s). In a second test, we kept a constant sampling frequency (12.5 Hz) and a constant updating interval (of 1 s) and then studied the effect of a broadcasting frequency of 4 Hz, 8 Hz, 12 Hz and 16 Hz. In the last test, we considered a constant updating interval (of 1 s) and a constant broadcasting frequency (16 Hz) and compared sampling frequencies of 26 Hz, 52 Hz, 104 Hz and 208 Hz. For each scenario, we configured the sensors with the RAMSMART firmware, equipped them with a (fully charged) GP Extra Lithium CR2025 3 V coin cell battery and connected them to a single laptop through BLE. We collected data until batteries were flat and we then compared the battery life of the three replicates across the scenarios.

### 2.5 Evaluation of data completeness and variation across sensors

When multiple sensors and/or high updating frequencies are used, we expected that a sufficiently high broadcasting frequency would be needed to ensure that all data is captured by the receiving device. We explored the effect of four different broadcasting frequencies (4 Hz, 8 Hz, 12 Hz and 16 Hz), combined with two different updating frequencies (once every second, and once every minute), on data completeness. The sampling frequency was kept constant at 12.5 Hz and the sensors were placed next to the receiving device (< 0.3 m distance). Each scenario was run for 12 h with 8 sensors, which were simultaneously connected to a single laptop. Missing data packets were identified based on the incremental identifiers of the data packets. The identifiers of the first and last received packet were used as reference, and all absent identifiers in between were considered missing data packets. For each sensor-hour combination, the number and percentage of missing data packets was determined and were used as a measure of data incompleteness.

Aside from missing data packets at the receiving side, we expected that sensors may also vary in the number of unique data packets they broadcast per hour, because of minor hardware differences. To study possible variation across sensors, we also compared the number of received data packets per hour for scenarios without missing data packets.

### 2.6 Showcases

To demonstrate RAMSMART in practice, we employed the system in three research trials. In these trials, experimental vaccine candidates against viral infections were evaluated in pigs, calves and sheep. Animals were challenged with an infectious disease (on day “0”) and monitored with RAMSMART from approximately 1 week before the challenge, until the end of the trial. In all trials, the sampling frequency was set to 12.5 Hz, the broadcasting frequency was set to 4 Hz, the updating frequency was set to approximately once every minute and the rolling average in the VeDBA calculation was based on 2 s. In addition to RAMSMART, in all trials various disease parameters were monitored on a daily base (starting from day −2) including rectal temperature and specific clinical signs using semi-quantitative severity scores for, among others, activity/alertness, appetite, respiration and disorders in limbs & locomotion. These scores were combined into a cumulative clinical score.

#### 2.6.1 Pig trial

**Figure 2:**
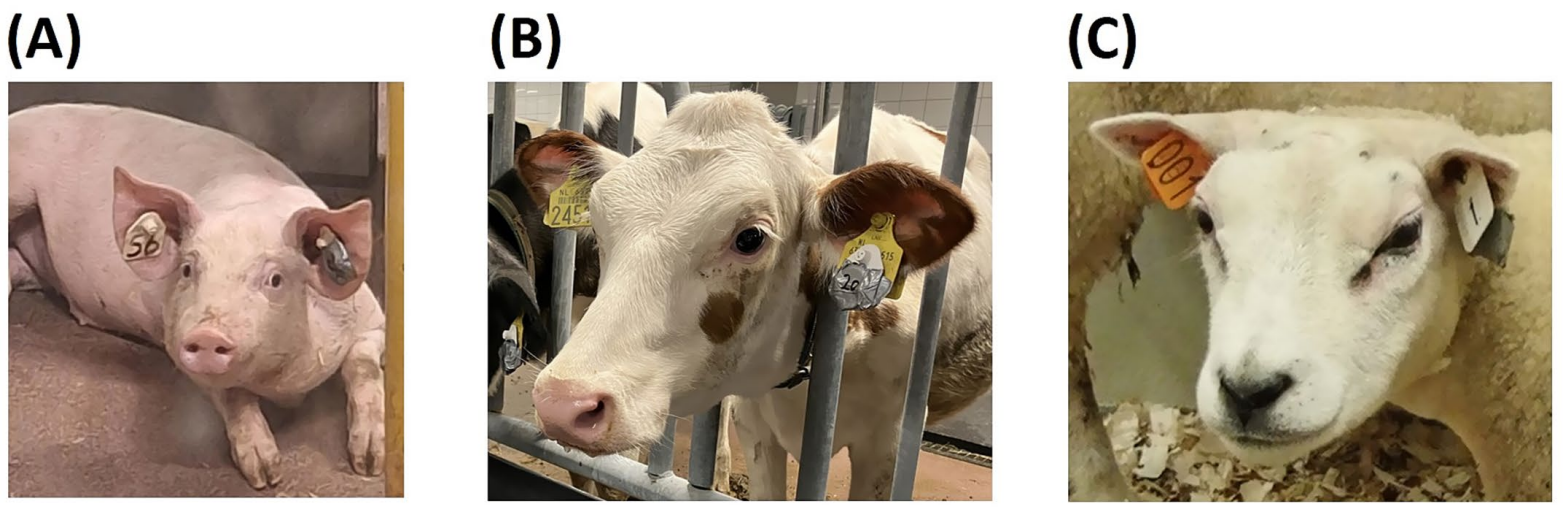
Attachment of accelerometers to the ear tag of animals of various species. Examples of **(A)** the pig trial, **(B)** the calf trial, and **(C)** the sheep trial.

**Figure 3:**
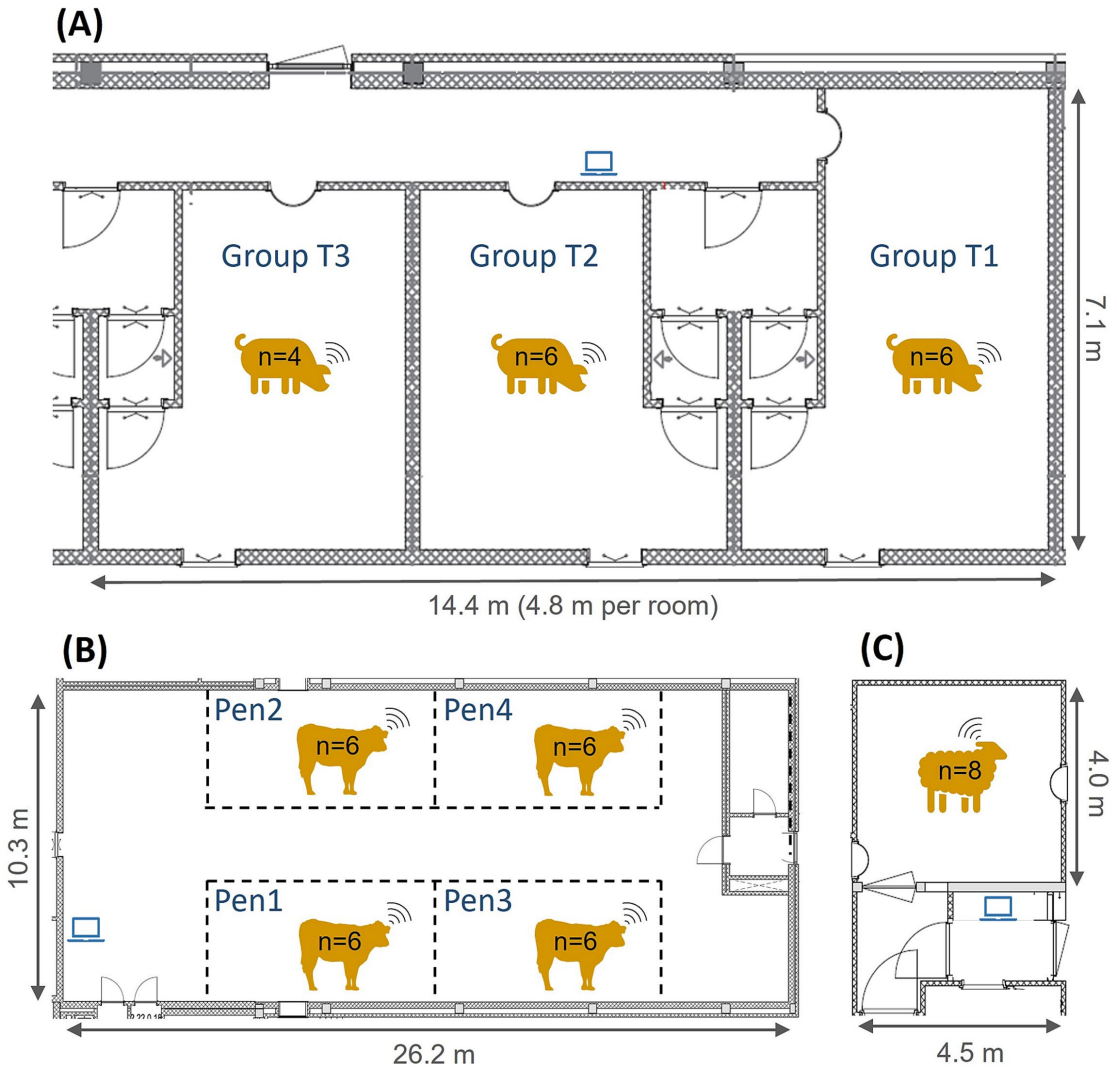
Set-up of RAMSMART in the pig, sheep, and calf trials. **(A)** Three rooms of 20 to 25 m^2^ each, with a single laptop for all rooms in the common corridor. **(B)** Room of 255 m^2^ in the calf trial, subdivided into four pens (each of 33 m^2^) with metal fences, and the laptop near the entrance of the room. **(C)** Example of one of the three rooms of 23 m^2^ in the sheep trial, with the laptop in the neighboring preparation room, where each room had a separate laptop.

In the pig trial, 16 female pigs (cross of Large White and Norwegian Landrace) of ~10 weeks old were challenged on October 29th, 2025 (day “0”) with genotype 2 African Swine Fever Virus (ASFV), strain Armenia ‘07. Pigs were assigned to three treatment groups. Four weeks prior to challenge, the groups either had received a candidate vaccine orally (T1, *n* = 6), the same candidate vaccine intramuscularly (T2, *n* = 6), or had been mock-vaccinated with a placebo (T3, *n* = 4). Eight days before the challenge (day −8), an accelerometer was attached to the ear tag of each pig (Figure 2A) and RAMSMART was run until 15 days after the challenge (day 15), or until individual humane endpoints were reached. The three treatment groups were housed in separate and adjacent rooms, each 20 to 25 m2, and a single laptop for receiving the data was placed in the corridor next to these rooms (Figure 3A).

#### 2.6.2 Calf trial

In the calf trial, 24 female Holstein calves of ~24 weeks old were challenged on July 9th, 2024 (day 0) with epizootic haemorrhagic disease virus (EHDV-8). Calves were assigned to three treatment groups of eight calves each, which had been mock-vaccinated with a placebo (T1) or had received vaccine 1 (T2) or 2 (T3) at six and two weeks prior to challenge. On day −6, an accelerometer was attached to the ear tag of each calf (Figure 2B) and RAMSMART was run until day 22. Calves were co-mingled in four pens, with two calves of each treatment per pen. Each pen was 8.2 m long and 4.0 m wide and all pens were in a single large room of 255 m2 (Figure 3B). A single laptop was placed in the corner of this room to receive the data.

#### 2.6.3 Sheep trial

In the sheep trial, three groups (T1, T2, and T3) of each eight Texelaar ewes of approximately one-year old were challenged on April 5th, 2024 (day “0”) with Bluetongue virus (BTV-3/Neth 2023). 4 weeks before the challenge, group T1 had been mock-vaccinated with a saline placebo, and groups T2 and T3 had received BTV-3 vaccine A and B, respectively. Seven days before the challenge (day −7), an accelerometer was attached to the ear tag of each sheep (Figure 2C) and RAMSMART was run until 20 days after the challenge (day 20). Each group was housed in a separate room of approximately 23 m2. For each group, a laptop for receiving the data was placed in the preparation room that was directly connected to the animal room (Figure 3C).

## 3 Results

### 3.1 Battery life

**Figure 4:**
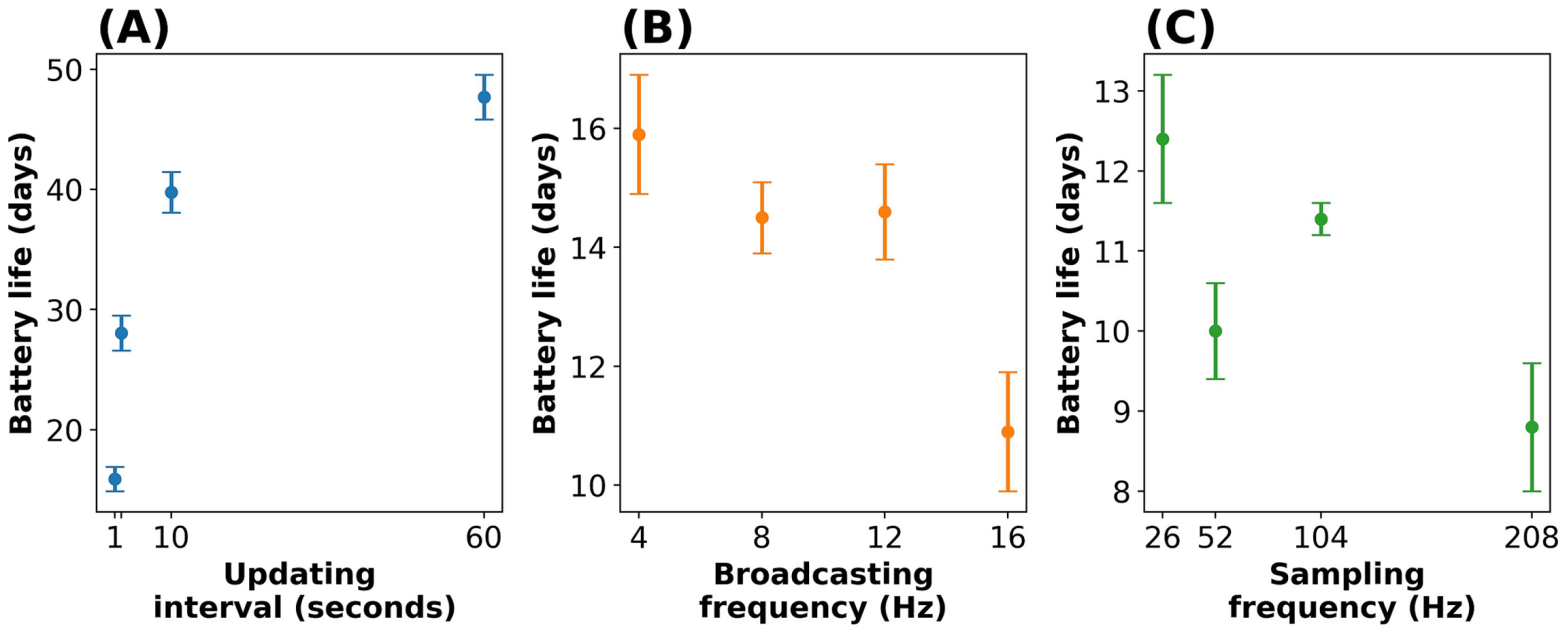
Battery life for different configurations, showing the mean of three replicates for each configuration, with error bars representing ± 1 standard deviation. **(A)** Effect of the updating interval, when the broadcasting frequency is 4 Hz and the sampling frequency is 12.5 Hz. **(B)** Effect of the broadcasting frequency, when the updating interval is once every second and the sampling frequency is 12.5 Hz. **(C)** Effect of the sampling frequency, when the updating interval is once every second, and the broadcasting frequency is 16 Hz.

A longer updating interval resulted in longer battery life (Figure 4A; Supplementary Table S1). When the sampling frequency was kept constant at 12.5 Hz, and the broadcasting frequency at 4 Hz, an updating interval of 1 second resulted in a battery life of 15.9 days (± 1.0 days), whereas an interval of 1 minute resulted in a battery life of 47.7 days (± 1.9 days). The observed relationship between the updating interval and battery life was not linear; namely, further increasing the updating interval resulted in diminishing returns in battery life.

Higher broadcasting frequencies resulted in shorter battery life (Figure 4B; Supplementary Table S1). When the sampling frequency was kept constant at 12.5 Hz, and the updating interval at 1 second, the battery life for a broadcasting frequency of 4 Hz was 15.9 days (± 1.0 days), which reduced to 10.9 days (± 1.0 days) for a broadcasting frequency of 16 Hz. The relationship also appeared not linear, but the trend was less convincing due to the larger variation across sensors relative to the mean.

Higher sampling frequencies also resulted in shorter battery life (Figure 4C; Supplementary Table S1). When the sampling updating interval was kept constant at 1 second, and the broadcasting frequency at 16 Hz, the battery life for a sampling frequency of 26 Hz was 12.4 days (± 0.8 days), which reduced to 8.8 days (± 0.8 days) for a sampling frequency of 208 Hz.

### 3.2 Data completeness and variation across sensors

When a sampling rate of 12.5 Hz and an updating interval of 1 minute were combined with a broadcasting frequency of 4 Hz, all data packets were successfully received (Table 1 for statistics across all sensors; Supplementary Table S2 for statistics per sensor). Even when the broadcasting frequency was reduced to 1 Hz, only on average 0.5 data packets per hour (< 1% of total) were lost. For a shorter updating interval of 1 second, higher broadcasting frequencies were needed to ensure that most data packets were received. For example, broadcasting frequencies of 4 Hz and 16 Hz resulted in on average 1814 (51%) and 152 (4.3%) data packets missing per hour, respectively.

**Table 1:** Number (N) of missing data packets per hour when different broadcasting frequencies and updating frequencies were used.

Broadcasting frequency	Update every second		Update every minute	
	N per hour, mean (SD)	N missing per hour, mean (SD)	N per hour, mean (SD)	N missing per hour, mean (SD)
1 Hz	532.2 (25.2)	2980.2 (131.8)	58.4 (2.2)	0.5 (0.7)
4 Hz	1797.7 (33.8)	1756.4 (120.2)	58.9 (2.2)	0 (0)
8 Hz	2682.0 (32.4)	850.4 (115.4)	58.9 (2.2)	0 (0)
12 Hz	3185.4 (81.6)	347.4 (56.9)	58.9 (2.2)	0 (0)
16 Hz	3381.5 (73.9)	151.6 (100.6)	58.9 (2.2)	0 (0)

Sampling frequency was kept constant at 12.5 Hz, and results were based on 8 sensors and 12 h per sensor (so 96 h in total).

**Figure 5:**
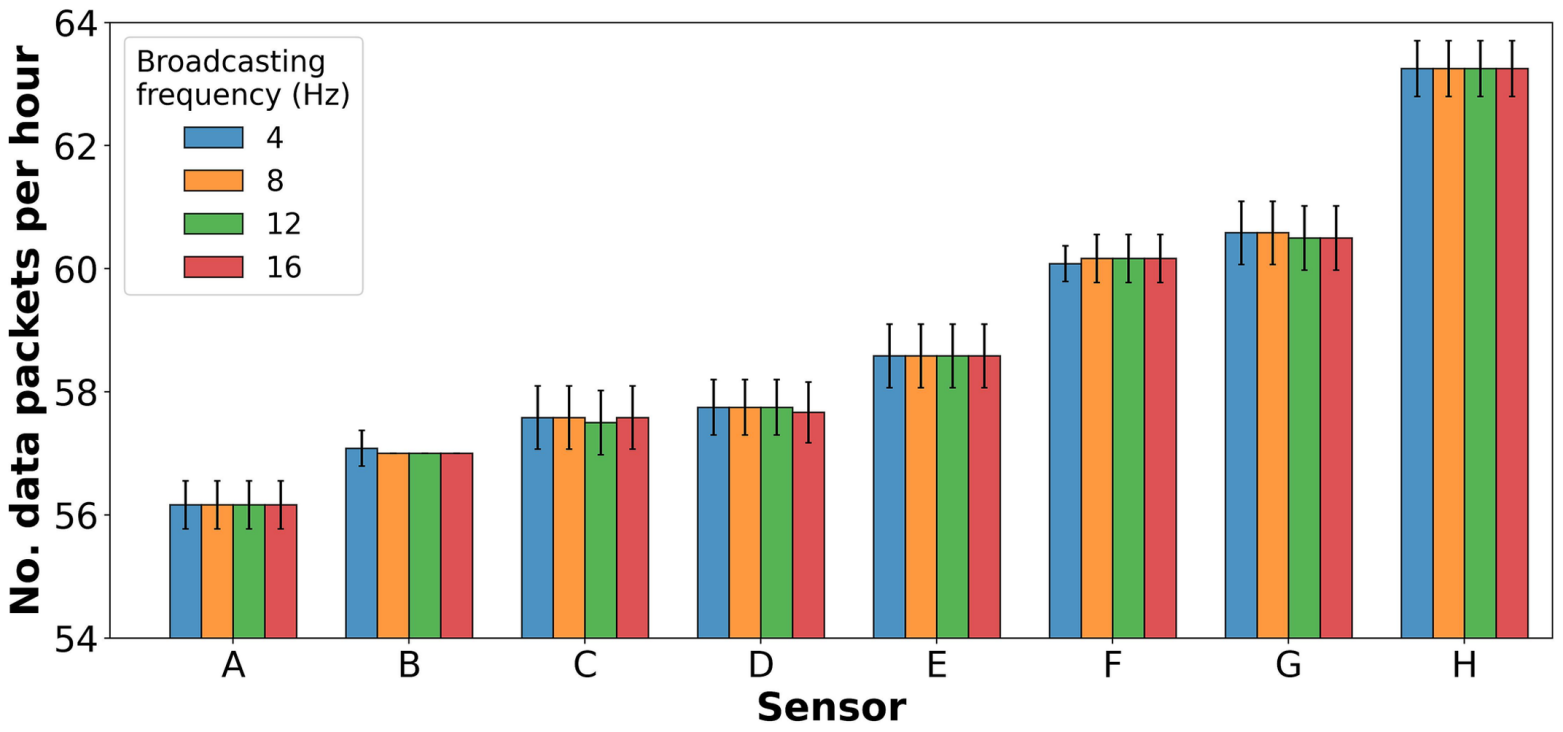
Variation in the mean number of data packets per hour across 8 sensors. Results are shown for the tested scenarios with all data packets successfully received (see Table 1 and Supplementary Table S2). These scenarios had a sampling frequency of 12.5 Hz, an updating frequency of approximately once every minute, and a broadcasting frequency of 4, 8, 12, or 16 Hz. Bars represent the mean for the 12 h per sensor, and error bars represent ± 1 standard deviation. Note that sensor B had a constant number of packets received (of 57 packets) across all 12 hours for broadcasting frequencies 8, 12, and 16 Hz and, therefore, had no standard deviation.

Aside from variation in the number of data packets that was lost on average, sensors systematically varied in how often they sent unique data packets, despite using the same configuration (Figure 5; Supplementary Table S2). For example, for a user-defined sampling frequency of 12.5 Hz and an updating interval of 1 minute, the eight sensors varied from approximately 56 to 63 data packets per hour. This variation seemed sensor specific, as illustrated by the systematic pattern across the different twelve-hour tests with different broadcasting frequencies (Figure 5).

### 3.3 Showcases

#### 3.3.1 Pig trial

In the pig trial, on average 1.3 data packets were missing per animal per hour across all collected data (2.2% of total). Group T2, which was housed in the room closest to the receiving device, had on average 0.64 (±0.05) packets missing per animal per hour. Groups T1 and T3, which were housed further away from the receiving device (Figure 3A), had on average 2.39 (±0.13) and 1.17 (±0.10) data packets missing per animal per hour, respectively. Across animals, the percentage of missing data packets for the entire monitoring period varied from 0.59 to 5.78% (Supplementary Table S3).

**Figure 6:**
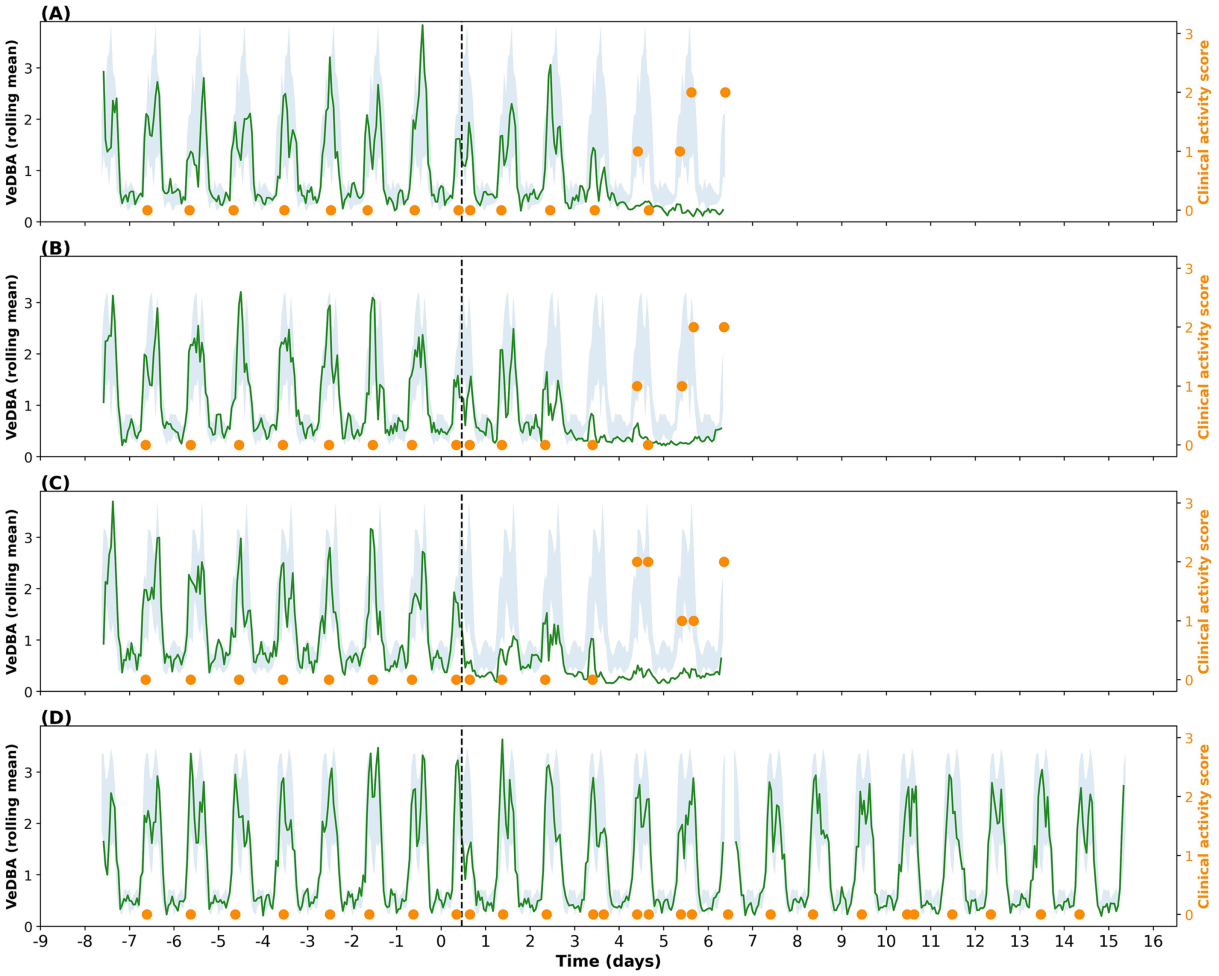
Example trends of the vectorial dynamic body acceleration (VeDBA) of individual pigs before and after a challenge with African swine fever virus. The challenge occurred on day 0 (dashed vertical line) and tick marks indicate 00:00 h of each day. The VeDBA (in green) is shown as a rolling mean of 3 h, and a reference range of “normal VedBA levels” (in blue) is shown based on the minimum and maximum levels for the corresponding hour of the individual on the days before the challenge. In addition, clinical activity scores based on human (bi-)daily observations (in orange) are shown. These clinical scores were defined as follows: (0) Normal, (1) Slow, still gets up on its own, without help, (2) Slow, gets up with some help, lies down quickly, and (3) Stays down, does not get up even after some pressure. **(A)** Example of a pig of the mock-vaccinated group T3, for which a humane endpoint was applied on day 6. **(B, C)** Example of two pigs that were part of the orally vaccinated group T1, for which humane endpoints were applied on day 6. **(D)** Example of a pig that was part of the intramuscularly vaccinated group T2.

RAMSMART provided valuable insights in the 24/7 activity patterns of the pigs. Each pig showed a circadian activity pattern, with high VeDBA-levels during the day, and low levels during the night (Figure 6, Supplementary Figure S1). A few days after the challenge, various animals showed a severe drop in sensor-based activity, with—for some animals—daytime VeDBA levels decreasing to less than 20% of their ‘normal’ activity levels. RAMSMART detected these deviations typically one to three days earlier than the traditional (bi-) daily human observations (Figures 6A–C, Supplementary Figure S1). Some animals showed no deviations in their ‘normal’ activity pattern over time, which was in line with the absence of clinical symptoms for those animals (e.g., Figure 6D).

**Figure 7:**
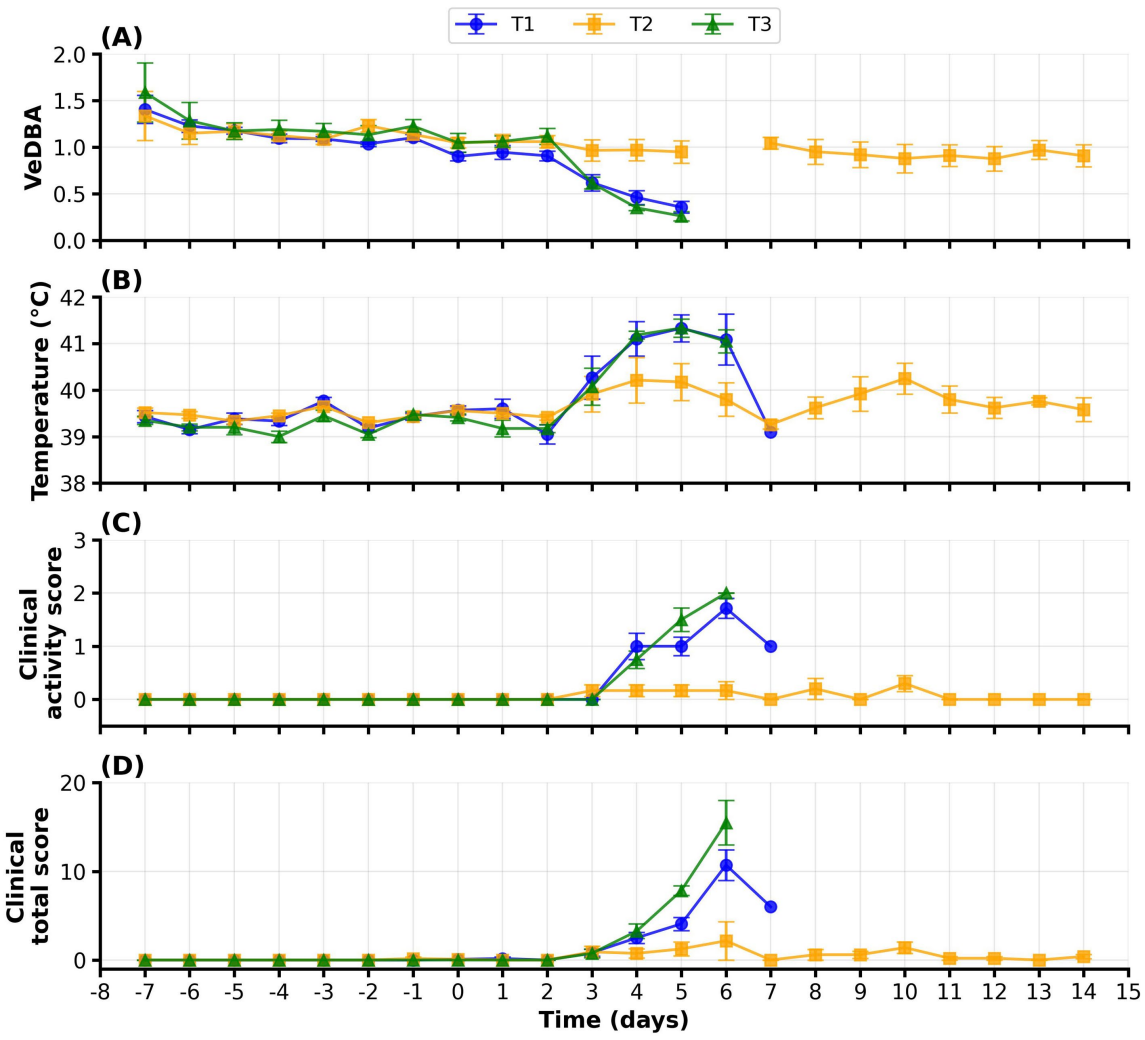
Daily monitoring results of pigs before and after a challenge (on day 0) with African swine fever virus. Three groups of eight pigs are compared: T1 and T2 received a candidate vaccine intramuscularly and orally, respectively, and T3 was mock-vaccinated. For group T1, humane endpoints were applied on day 6 (*n* = 5), day 7 (*n* = 1), and day 11 (*n* = 2). For group T2, a humane endpoint was applied on day 6 (*n* = 1), while the other animals were kept until the end of the study (*n* = 5). For group T3, humane endpoints were applied on day 5 (*n* = 2) and day 6 (*n* = 2). For each group, the mean and standard deviation (as error bar) per day are shown. **(A)** The vectorial dynamic body acceleration as obtained with RAMSMART. **(B)** The rectal temperature, where >40° C° was considered fever. **(C)** The clinical activity score. **(D)** The cumulative clinical score, as a sum of, among others, activity/alertness, appetite, and disorders in limbs & locomotion.

From day 3 onwards, the mean daily VeDBA was significantly lower in the mock-vaccinated group T3 and the orally vaccinated group T1 than in the intramuscularly vaccinated group T2 (Figure 7A). On day 3, animals also developed fever (Figure 7B). From day 4 onwards the mean body temperature, the mean clinical activity score, and the mean total clinical score were significantly higher in T3 and T1 than in T2 (Figures 7B–D).

#### 3.3.2 Calf trial

In the calf trial, one sensor stopped broadcasting data on day 10 for an unknown reason. Aside from this single sensor, the data obtained with RAMSMART showed reasonable completeness, with on average 1.2 data packets missing per sensor per hour (< 2% of total) across all collected data. Notably, the mean number of missing data packets per hour differed across the four pens. With 6 sensors per pen, the mean number of missing data packets per hour was 0.41 (± 0.03), 1.06 (± 0.05), 1.22 (± 0.05) and 2.00 (± 0.07) for Pen1, Pen2, Pen3 and Pen4, respectively (see Figure 3B for pen set-up). Across animals, the percentage of missing data packets for the entire monitoring period varied from 0.48 to 4.17% (Supplementary Table S3).

**Figure 8:**
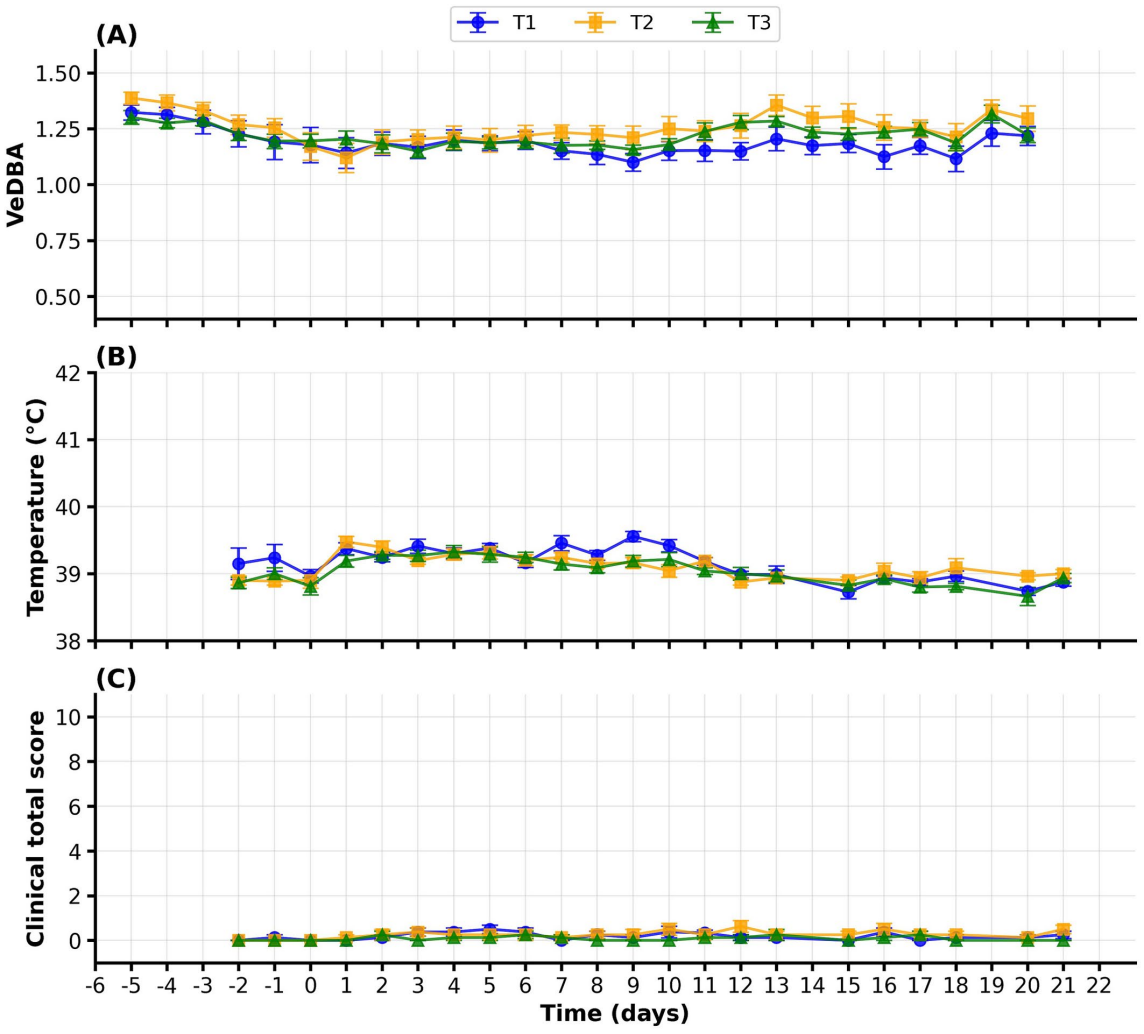
Daily monitoring results of calves before and after a challenge (on day 0) with epizootic haemorrhagic disease virus. Three groups of eight sheep are compared: T1 was a placebo group and T2 and T3 received vaccine 1 and 2, respectively. For each group, the mean and standard deviation (as error bar) per day are shown. **(A)** The vectorial dynamic body acceleration as obtained with RAMSMART. **(B)** The rectal temperature, where >39.5 °C was considered fever. **(C)** The cumulative clinical score, as a sum of, among others, activity/alertness, appetite, and disorders in limbs & locomotion. Note that clinical activity scores were all equal to zero (not shown in figure).

Calves showed a circadian activity pattern, with higher activity levels during the day, and low levels during the night (Supplementary Figure S2). The daily VeDBA showed no significant differences in VeDBA levels over time nor between the treatment groups (Figure 8A, Supplementary Figure S2). This observation was in line with the absence of fever and absence of other clinical symptoms (Figures 8B–C, Figure 8C).

#### 3.3.3 Sheep trial

Due to start-up issues when using RAMSMART for the first time in the sheep trial (namely, laptops going on standby), data from group T3 was missing for the days prior to day −2, and data for group T1 was missing on parts of day −2 and day −1. However, when RAMSMART was effectively running, data was highly complete, with on average less than 0.01 data packet missing (<0.02%) per sensor per hour. Across animals, the percentage of missing data packets for the entire monitoring period varied from 0 to 0.23% (Supplementary Table S3).

**Figure 9:**
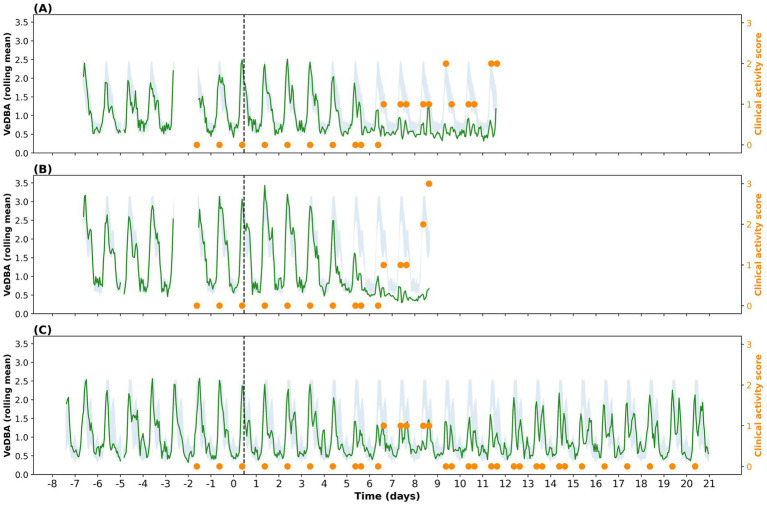
Example trends of the vectorial dynamic body acceleration (VeDBA) of individual sheep before and after a challenge with bluetongue virus. The challenge occurred on day 0 (dashed vertical line) and tick marks indicate 00:00 h of each day. The VeDBA (in green) is shown as a rolling mean of 3 h, and a reference range of “normal VedBA levels” (in blue) is shown based on the minimum and maximum levels for the corresponding hour of the individual on the days before the challenge. **(A, B)** Examples of two non-vaccinated sheep of the placebo group T1, for which humane endpoints were applied on day 11 and 8. **(C)** Example of a vaccinated sheep of treatment group T2.

**Figure 10:**
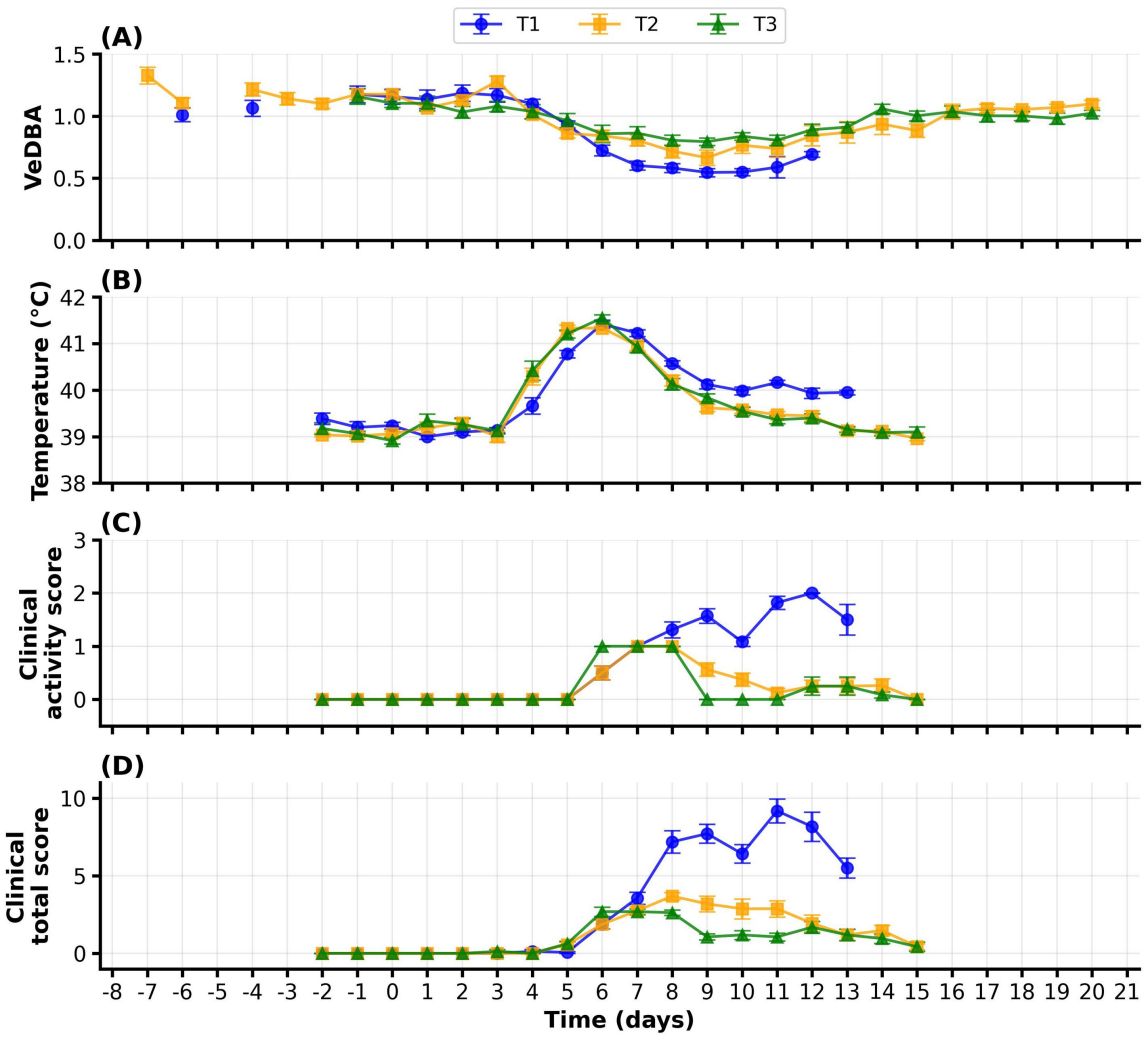
Daily monitoring results of sheep before and after a challenge (on day 0) with bluetongue virus. Three groups of eight sheep are compared: T1 was a placebo group and T2 and T3 received vaccine A and B, respectively. For group T1, humane endpoints were applied on day 8 (*n* = 1), day 9 (*n* = 1), day 11 (*n* = 2), day 12 (*n* = 2), and day 13 (*n* = 2). For groups T2 and T3, humane endpoints were applied on day 14 (*n* = 1 for each group). For each group, the mean and standard deviation (as error bar) per day are shown. **(A)** The vectorial dynamic body acceleration as obtained with RAMSMART. **(B)** The rectal temperature, where >40 °C was considered fever. **(C)** The clinical activity score. **(D)** The cumulative clinical score, as a sum of, among others, activity/alertness, appetite, and disorders in limbs & locomotion.

Each sheep showed a circadian activity pattern, with high VeDBA-levels during the day, and low levels during the night (Figure 9, Supplementary Figure S3). From day 4 post BTV challenge onwards, daytime activity levels dropped, with some sheep – of particularly the placebo group – showing a severe decrease to less than 25% of their ‘normal’ VeDBA from before the challenge (Figure 9A, Figure 9B). The placebo group (T1) showed the largest drop of on average approximately 50%, i.e., from a mean VeDBA of 1.2 in days −2 to 3, to a mean VeDBA of less than 0.6 in days 6 to 10 (Figure 10A). The treatment group with the vaccine B (T3) showed the mildest drop of approximately 30%, i.e., from a mean VeDBA of 1.1 in days −2 to 3, to a mean VeDBA of 0.8 in days 6 to 10. The observed drops in VeDBA, and the less severe drop for the vaccinated animals, were in line with the onset of fever, and with the increase in cumulative clinical scores (Figure 10). For several animals, RAMSMART detected drops in activity 0.5 to 1.5 days before the rise in clinical depression scores (e.g., Figure 9). This earlier detection of deviations was also visible at group level, e.g., for groups T1 and T2 the mean VeDBA on day 5 was significantly lower than on previous days (Figure 10A), whereas clinical activity scores for these groups started to rise significantly on day 6 (Figure 10C). In addition, for some animals that showed a drop in activity but later recovered, the observed period with decreased activity levels based on RAMSMART was longer than what was recorded based on clinical depression scores (e.g., Figure 9C).

## 4 Discussion

In this study, we developed a system for real-time automated monitoring of animal activity in research trials (RAMSMART). We evaluated how battery life and data completeness were influenced by the updating interval, the broadcasting frequency and the sampling interval. In addition, we showcased the application of RAMSMART in three infectious disease studies in pigs, calves and sheep.

In the animal trials, we have demonstrated the added value of RAMSMART to detect activity deviations up to 3 days earlier than based on traditional (bi-) daily observations. In addition, for some animals in the sheep study, RAMSMART also reported a longer drop in activity levels than what was detected based on human observations. This is in line with earlier findings (9) and could be due to a habituation effect in the human observations, the effect of having different human observers over time, a snapshot-effect in the human observations, or a difference between VeDBA measurements and the definition of the clinical scores. Moreover, RAMSMART helped to better quantify the observed individual behavioral deviations, by expressing the decrease in activity as a continuous measure—namely a percentage of an animal’s normal baseline—instead of using a 4-point score for visual observations. Thereby, RAMSMART facilitated a more detailed evaluation of the effect of the treatment at the individual animal level, and at the group level (comparing treatment and control groups). Last, the real-time data transfer and automated visualisations allowed the involved project leaders to timely interpret the results, which—in some cases—aided in the decision on applying humane endpoints.

RAMSMART meets most of the requirements that are important for continuous monitoring of animal activity in an experimental research environment. First, the system is generalizable across multiple species. So far, we have used Move sense (or equivalent) accelerometers in pigs, sheep, calves and chickens (unpublished data). Given the weight of the sensor (10 grams only), and the rule of thumb that a wearable sensor should not weigh more than 5% of an animal’s body weight (29), RAMSMART could be used for animals as small as 200 grams. Species-specific adjustments can be made if desired (e.g., in terms of sampling frequency or the static component correction in the calculation of VeDBA) but are not essential.

Second, the battery life of close to 7 weeks when using an updating interval of 1 minute, a broadcasting frequency of 4 Hz, and a sampling frequency of 12.5 Hz (Figure 4A), is sufficient for many experiments. Thus, using such settings, there is no need for repeated handling of the animals to replace batteries. When a higher resolution of measurements is needed, e.g., if one is interested in the VeDBA per second instead of per minute, batteries will last a couple of weeks (Figure 4A) and—depending on the length of the intended monitoring period — may need to be replaced. Higher sampling frequencies can also be used, again at the cost of shorter battery life (Figure 4C). In our experience, a sampling frequency of 12.5 Hz is more than sufficient to monitor general VeDBA patterns, particularly when using a rolling average of multiple hours (28). For experiments longer than 7 weeks, one could further increase battery life by lengthening the updating interval, albeit with diminishing returns (Figure 4A) and at the cost of lower data resolution. Alternatively, one could reduce the broadcasting frequency, which may result in data incompleteness, likely depending on the number of sensors used, the combination with the updating interval, and the possible use of multiple receivers.

Third, the system is relatively plug-and-play. Once the sensors and receiving laptop have been configured, the only steps required prior to each experiment are to replace the battery of each sensor, to attach a sensor to each animal, to place the laptop in or near the animal room, and to run the receiver component of RAMSMART. Thus, in contrast to various other technologies, RAMSMART does not require laborious installation (e.g., of cameras, beacons, or antennae) per experiment.

Fourth, identification of individuals under group housing is guaranteed using the sensor’s Bluetooth address linked to the animal identifier. Thereby, an individual’s VeDBA pattern can be readily linked to other physiological, immunological, and behavioral observations.

Fifth, the use of automated data processing with edge computing limits data storage requirements and allows real-time interpretation of results. For example, in our scenarios with an updating interval of once per minute, RAMSMART produced approximately 0.1 Mb of data per animal per day. In addition, (near) real-time visualization of individual and group VeDBA trends were readily implemented (examples are shown in Figures 6–10). One could further expand the automated analyses, for example to send an alert to the involved researchers and/or biotechnicians when an animal deviates substantially (e.g., < 40% or >160%) from its normal VeDBA level. Thereby, RAMSMART could be effectively used as an early warning system.

Last, due its durability, dust profess and water resistance to 30 meters (allowing for wet surface disinfection with adequate active substance), the sensor can be re-used in multiple experiments. This re-usability, together with reasonable purchasing costs of 100 euros per sensor and 0.6 euros per battery, limited data storage costs, and no need for paying (yearly) licenses for commercial products, makes RAMSMART a relatively affordable system as compared to other sensor-based activity monitoring systems.

While testing and showcasing RAMSMART, we also identified some limitations. First, we found variation across sensors in the number of data packets they broadcasted, despite using the same configuration (Table 1, Figure 5). Since this variation was sensor-specific, we expect this is caused by the hardware and, for example, due to slight differences in calibration. While this may affect comparisons across (groups of) animals slightly, it will not influence the main purpose of detecting changes in activity patterns within animals over time.

A second limitation of RAMSMART is the possibility of losing data packets, which is influenced by several aspects related to communication through BLE. The communication range of BLE is relatively small, which may cause data incompleteness if sensors are too far from the receiving device. This limitation is demonstrated by our findings. In the sheep trial, with a distance of < 5 m and a wall between animals and the laptop (Figure 3C), data were highly complete (< 0.02% missing data packets per sensor per hour). In the calf trial with the larger animal room (Figure 3B), the number of missing data packets increased across pens with increasing distance. For example, Pen1 had on average 0.41 (± 0.03) missing data packets per animal per hour and had a maximum distance of ~13 m between the animals and the laptop, whereas Pen4 had on average 2.00 (± 0.07) missing data packets per animal per hour and had a maximum distance of ~25 m between the animals and the laptop. Similarly, in the pig trial, the rooms with the largest distance with the receiving device, and walls in between, had most data missing (though still <6% of all packets was missing). When analyzing the VeDBA as a rolling mean of 3 h, we expect that missing <6% of all packets does not affect the interpretation of activity time series. Nevertheless, based on our findings, we recommend limiting the distance between sensors and receiving device to < 20 m, and limit the number of walls in between where possible.

Packets may be lost due to limited communication range, and they may also be lost due to BLE advertising being inherently probabilistic. By increasing the broadcasting frequency, the probability increases that a (unique) packet is captured during a scan window. However, too high frequencies increase the risk of collision (i.e., multiple devices trying to send packets simultaneously on the same frequency channel) and may lead to congestion (i.e., the total traffic overwhelming the channel capacity). Currently, in our default settings with an updating interval of 1 minute and a broadcasting frequency of 4 Hz, each packet is broadcasted roughly 240 times (four times per second, for 60 s) and only needs to be received once. Thus, there is substantial redundancy. We observed that, for 8 sensors and a single laptop receiving the data, this redundancy was sufficient to receive all data packets (Table 1). When 24 sensors were connected to a single receiving laptop, as in the calf trial, less than 2% of data packets were missing (which was in part due to distance, as discussed above). For future work, it would be valuable to evaluate the balance between the number of sensors, broad-casting frequencies, updating intervals, data completeness and battery life in more detail.

**Figure 11:**
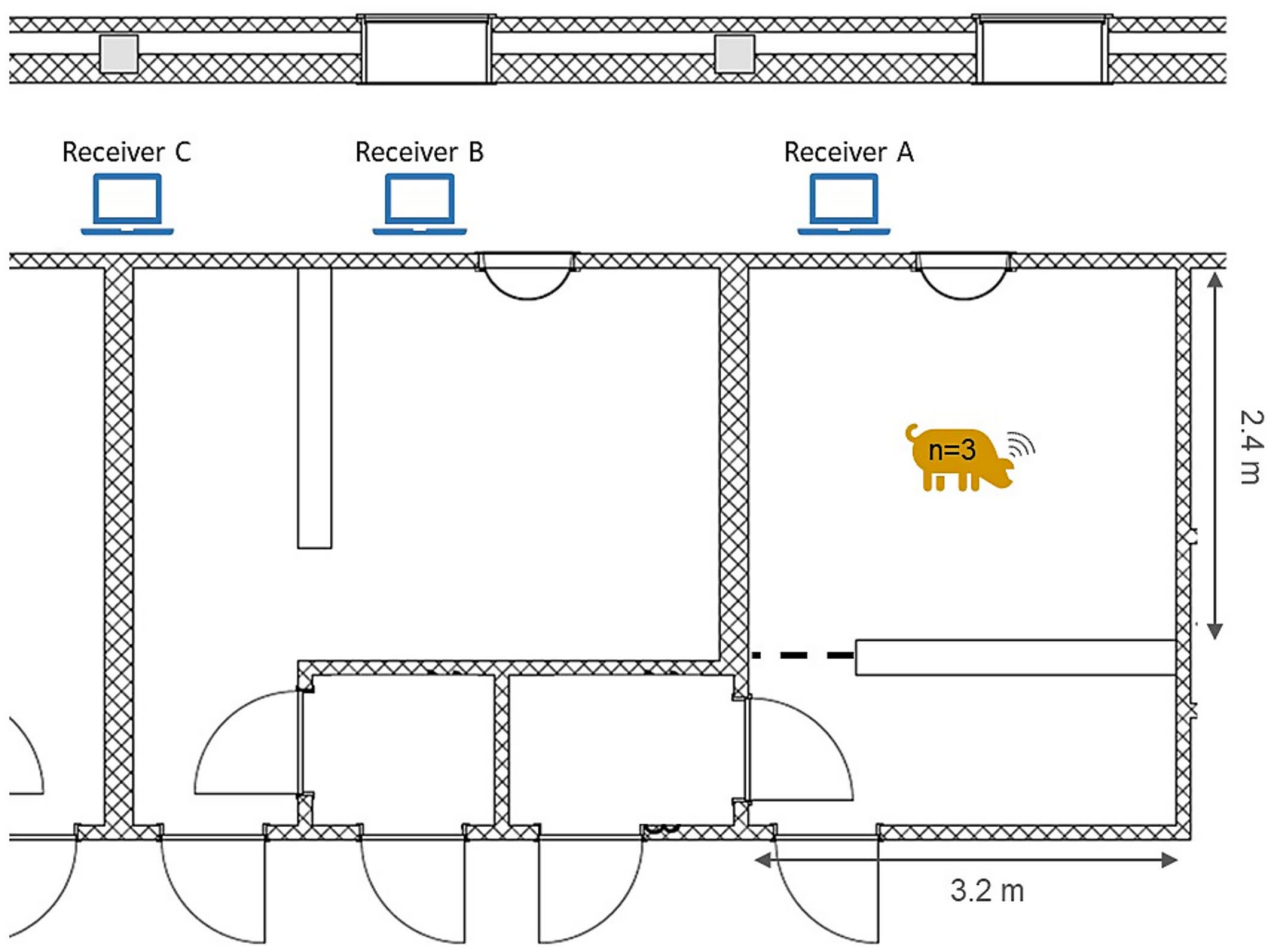
Set-up of a multi-receiver test in a group of three pigs with three receivers (A, B, and C) positioned in the corridor outside of the animal room. Pigs were kept for training purposes and not challenged with a disease.

To improve data completeness, multi-receiver set-ups could help to cover larger distances and prevent collision and congestion (30). We explored the added benefit of using multiple receivers in a test-case with three pigs used for training purposes. We monitored the VeDBA of these pigs with RAMSMART for 3 days with three receivers (Figure 11). As expected, receiver A, which was closest to the animal room, showed highest completeness with only 10 data packets (0.08%) missing, followed by receiver B with 88 data packets (0.70%) missing. Receiver C, which was furthest away, had 134 data packets (1.07%) missing (Table 2). By combining receivers B and C, the number of lost data packets was reduced to 65 (0.52%). Combining receiver A with receiver(s) B and C, however, did not help to improve data completeness, as the 10 data packets that were not captured by receiver A, were also not captured by receivers B and C. Thus, while multi-receiver set-ups can help to limit the number of lost data packets, they may not be able to achieve 100% data completeness. In future, it would be valuable to test multi-receiver set-ups in more detail, considering both distance and collision and congestion.

**Table 2:** Data completeness for a multi-receiver test case with three pigs monitored for 3 days with three receivers.

Receiver(s)	N broadcasted	N missing	Percentage missing (%)
A	12,548	10	0.08
B	12,548	88	0.70
C	12,548	134	1.07
A + B	12,548	10	0.08
A + C	12,548	10	0.08
B + C	12,548	65	0.52
A + B + C	12,548	10	0.08

All receivers were positioned outside the animal room and varied in distance from the animal room (see Figure 11). Numbers are shown for all three pigs combined.

While RAMSMART is currently developed to monitor animal activity, it also offers opportunities to expand towards monitoring of physiological parameters. Namely, the Movesense sensor also includes a one-channel ECG and a temperature sensor. Debruyne et al. (31) demonstrated that the Move sense sensor can be used for heart rate monitoring in cattle. By shaving the animals’ thorax and using a chest strap, they obtained good agreement with a widely used veterinary sensor (with a Pearson correlation coefficient of 0.98). At the same time, they demonstrated that the system is not promising for rectal temperature monitoring, which is not surprising given that it measures the internal temperature of the Movesense device. Extending RAMSMART to include heart rate monitoring would be interesting in near future, though it will come at the cost of reduced battery life and a more complex set-up. In addition, the use of a chest strap may not be possible in all species and the potential negative impact on the animal’s health and welfare should be well considered.

Aside from monitoring overall activity, the accelerometer data (as well as gyroscope and magnetometer data, which can also be obtained from the Move sense sensor) could also be used to detect specific behaviors, such as feeding, drinking or social behaviors. Many studies have demonstrated the use of accelerometer data to detect specific behaviors, for example when combined with machine learning classification models (13, 21, 22, 32). However, these models often require species-specific (and preferably environment-specific) training based on manually annotated data. Moreover, while classification models tend to achieve high prediction accuracies (of, e.g., > 95%) for relatively simple and frequent behaviors, more complex and less frequent behaviors, such as drinking or grooming behaviors, tend to show lower accuracies (e.g., < 80%). This is partly due to a lack of sufficient data to train the classification models, but also due to a larger variety in acceleration patterns for such complex behaviors. Moreover, it depends on the behavior of interest what position of the sensor is best. For example, while feeding behavior might be best predicted with neck-, jaw and ear-mounted sensors, the prediction of lying versus standing behavior may be easiest with leg-mounted sensors (22). Thus, adapting RAMSMART to monitor specific behaviors will imply losing (part of the) generalizability, and will decrease battery life. Uploading a pretrained classification model to the sensors is likely most promising in terms of battery life, although this will still use more battery than the current VeDBA calculations. For the sake of simplicity and battery life, we further recommend to limit the size of data packets below 26 bytes such that they can be sent through the Bluetooth advertisement protocol, without initiating a connection between sensors and receiving device. Given the limited generalizability and uncertain battery life, other technologies, such as an RFID system with antennae at drinkers/feeders, may be more suitable for long-term automated monitoring of specific behaviors across various species.

The system that we presented here, RAMSMART, together with other (sensor) data streams, could also be integrated into more general digitalization approaches, such as digital twins [for example, (33)]. In such a digital twin, RAMSMART would act as a “software sensor layer”, transforming raw data into interpretable variables). Thereby, it could help to keep track of a continuously updated, predictive model of an individual animal’s health. In general, the routine sensor-based tracking of animal behaviors in research trials helps to improve our understanding of the effect of experimental treatments, e.g., disease progression, and to refine experiments in the context of the 3Rs.

## 5 Conclusion

We developed a system for real-time automated multi-species monitoring of animal activity in research trials (RAMSMART). RAMSMART consists of a lightweight accelerometer that collects raw acceleration data, computes the VeDBA as a measure of activity and broadcasts this VeDBA through BLE to a receiving device. When using the VeDBA per minute as output, RAMSMART has a battery life of close to 7 weeks with reasonably good data completeness. The obtained data can aid researchers and biotechnicians with monitoring animal welfare and improving the assessment of an experimental treatment’s effects, as we demonstrated in three showcases in pigs, calves and sheep. In these showcases, RAMSMART improved the quantification of the onset, duration and severity of deviations in activity from each animal’s normal baseline. Overall, RAMSMART provides a low-invasive, affordable, re-usable, generalizable and plug-and-play approach for long-term real-time automated activity monitoring in animal research trials.

## Data Availability

The raw data supporting the conclusions of this article will be made available by the authors, without undue reservation.

## Footnotes

2
https://git.wur.nl/nlas-asg/ramsmart
