# Supplementary material for: RAMSMART: a low-invasive system for real-time automated multi-species monitoring of livestock activity in research trials
Source: Front Vet Sci. 2026 Jun 22;13:1830138. doi: 10.3389/fvets.2026.1830138 (PMC13333429; doi:10.3389/fvets.2026.1830138)
Supplement: Supplementary file 2 [file Supplementary_file_2.docx]

**
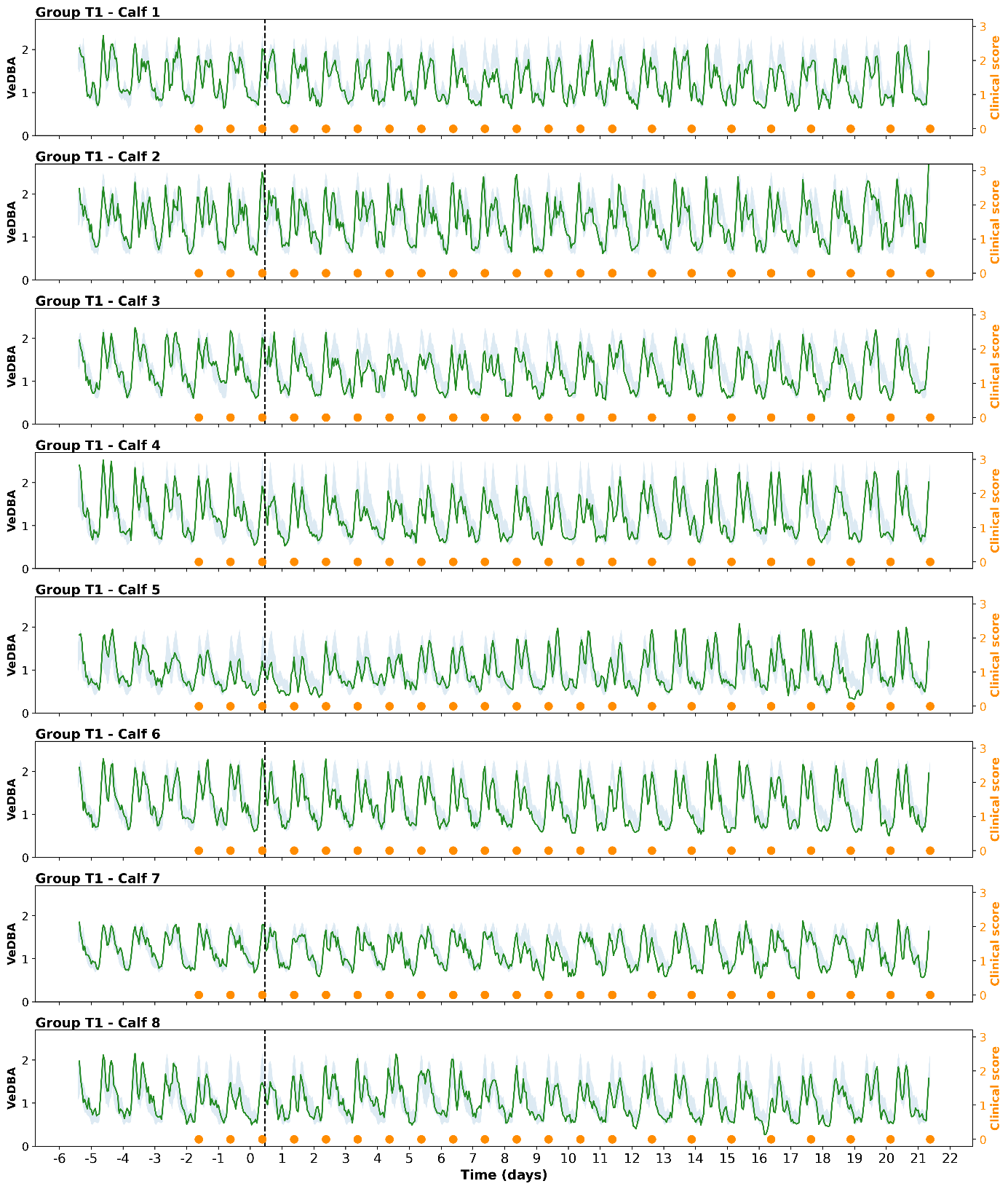
**

**Supplementary Figure S2.** Time series of the vectorial dynamic body acceleration (VeDBA) of individual calves before and after a challenge with epizootic haemorrhagic disease virus. The challenge occurred on day 0 (dashed vertical line). The VeDBA (in green) is shown as a rolling mean of 3 hours, and a reference range of ‘normal VedBA levels’ (in blue) is shown. In addition, clinical activity scores based on human (bi-)daily observations (in orange) are shown. These clinical scores were defined as follows: (0) Normal, (1) Slow, still gets up on its own, without help, (2) Slow, gets up with some help, lies down quickly, and (3) Stays down, doesn’t get up even after some pressure.

**
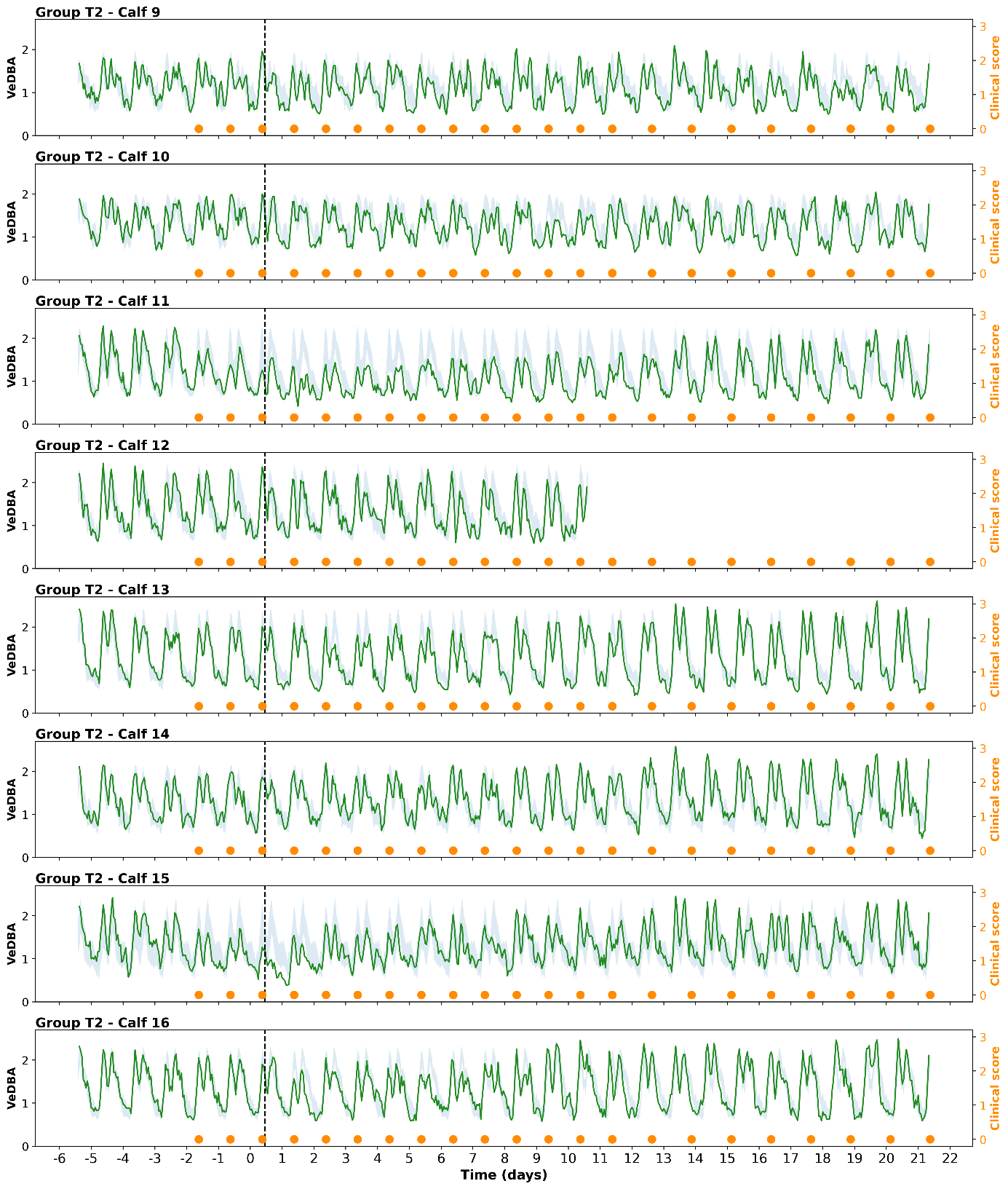
**

**Supplementary Figure S2.** (continued)

**
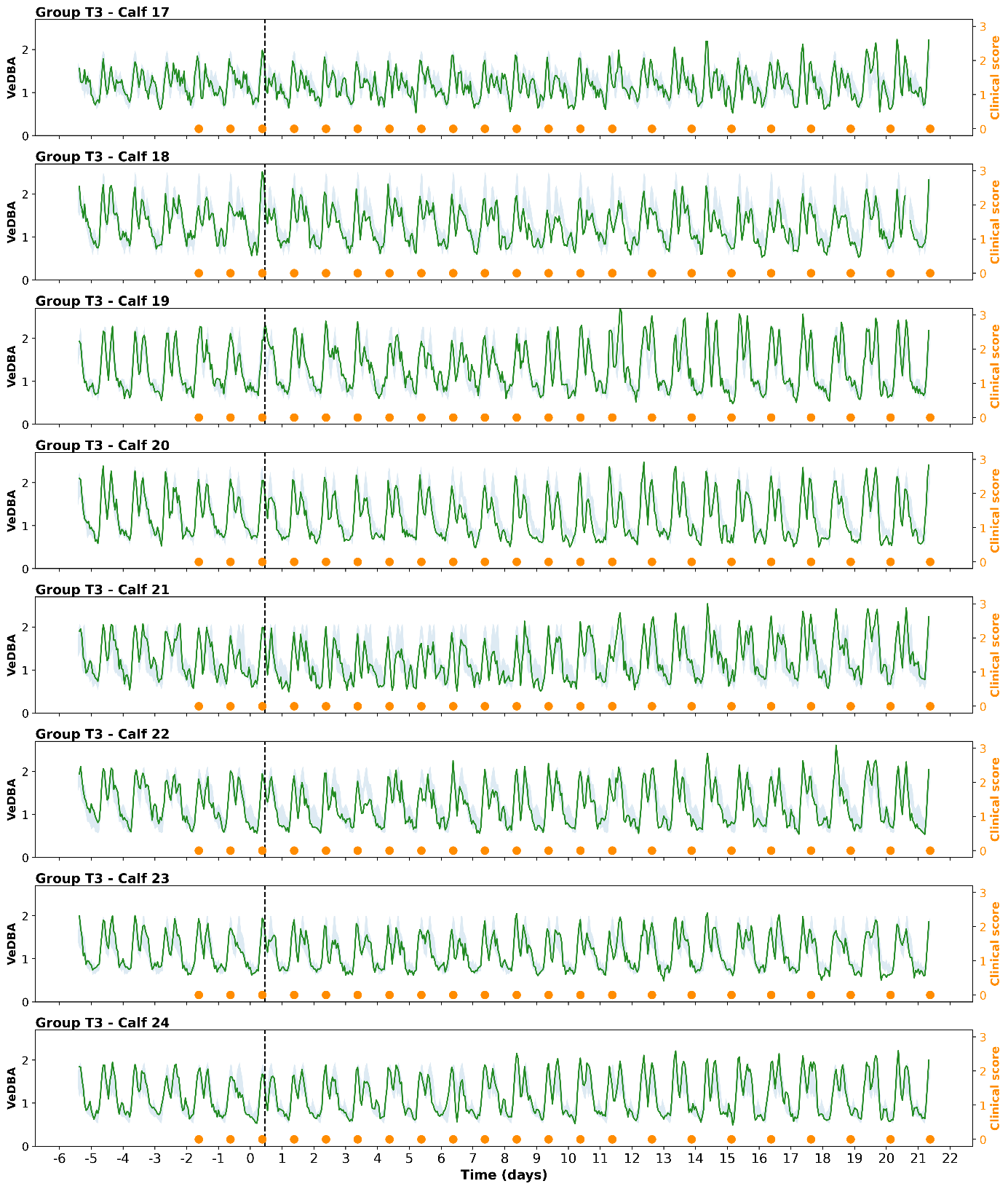
Supplementary Figure S2.** (continued)
