# Supplementary material for: RAMSMART: a low-invasive system for real-time automated multi-species monitoring of livestock activity in research trials
Source: Front Vet Sci. 2026 Jun 22;13:1830138. doi: 10.3389/fvets.2026.1830138 (PMC13333429; doi:10.3389/fvets.2026.1830138)
Supplement: Supplementary file 3 [file Supplementary_file_3.docx]

**
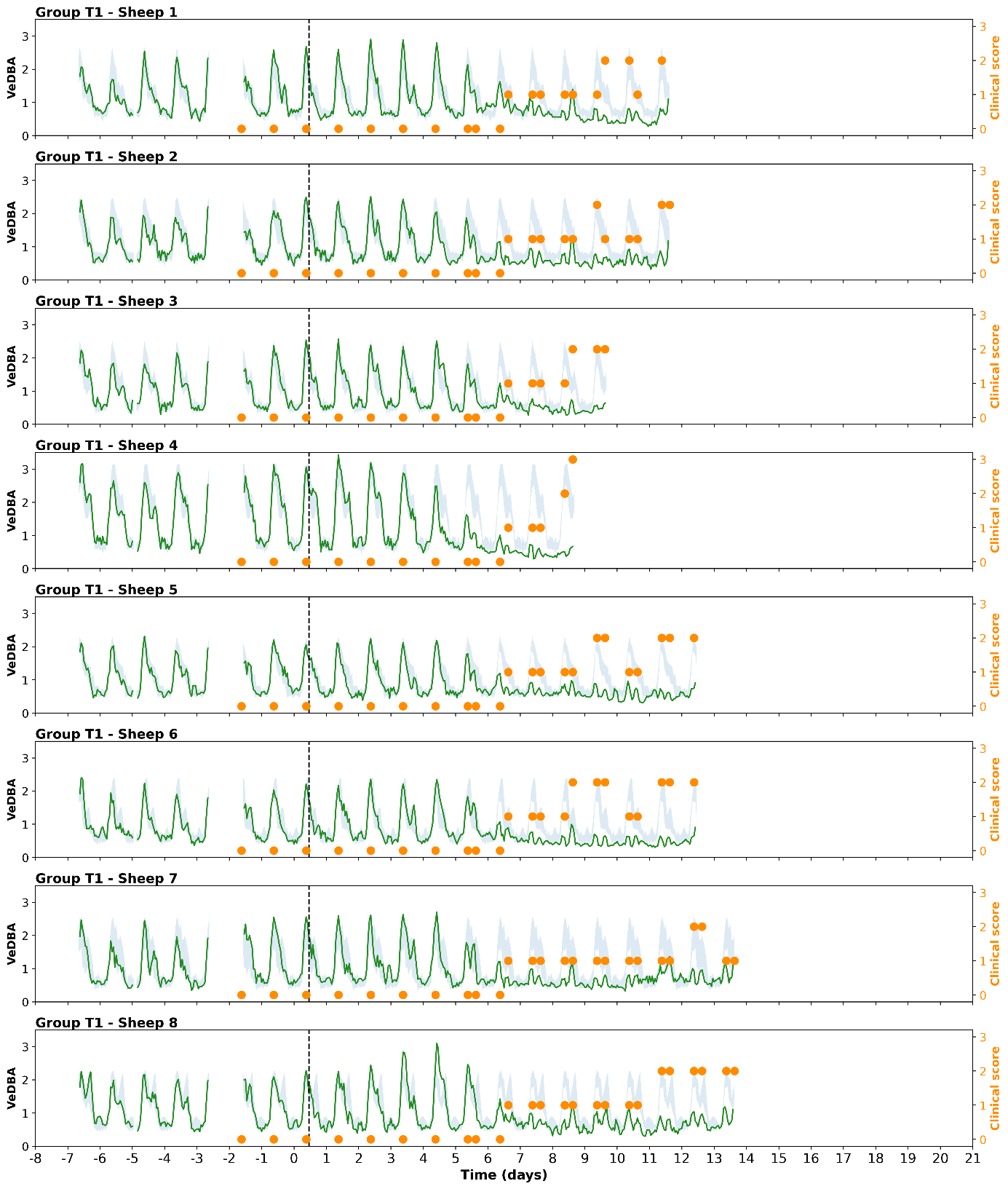
**

**Supplementary Figure S3.** Time series of the vectorial dynamic body acceleration (VeDBA) of individual sheep before and after a challenge with Bluetongue virus. The challenge occurred on day 0 (dashed vertical line). The VeDBA (in green) is shown as a rolling mean of 3 hours, and a reference range of ‘normal VedBA levels’ (in blue) is shown. In addition, clinical activity/depression scores based on human (bi-)daily observations (in orange) are shown. These clinical scores were defined as follows: (0) Alert, normal behavior, (1) Less active, less alert, (2) Lethargic, stays lying down longer or lies down more quickly, isolates itself, only responds after stimulation, and (3) Very lethargic, absent, does not stand up spontaneously, shows little to no response to stimuli.

**
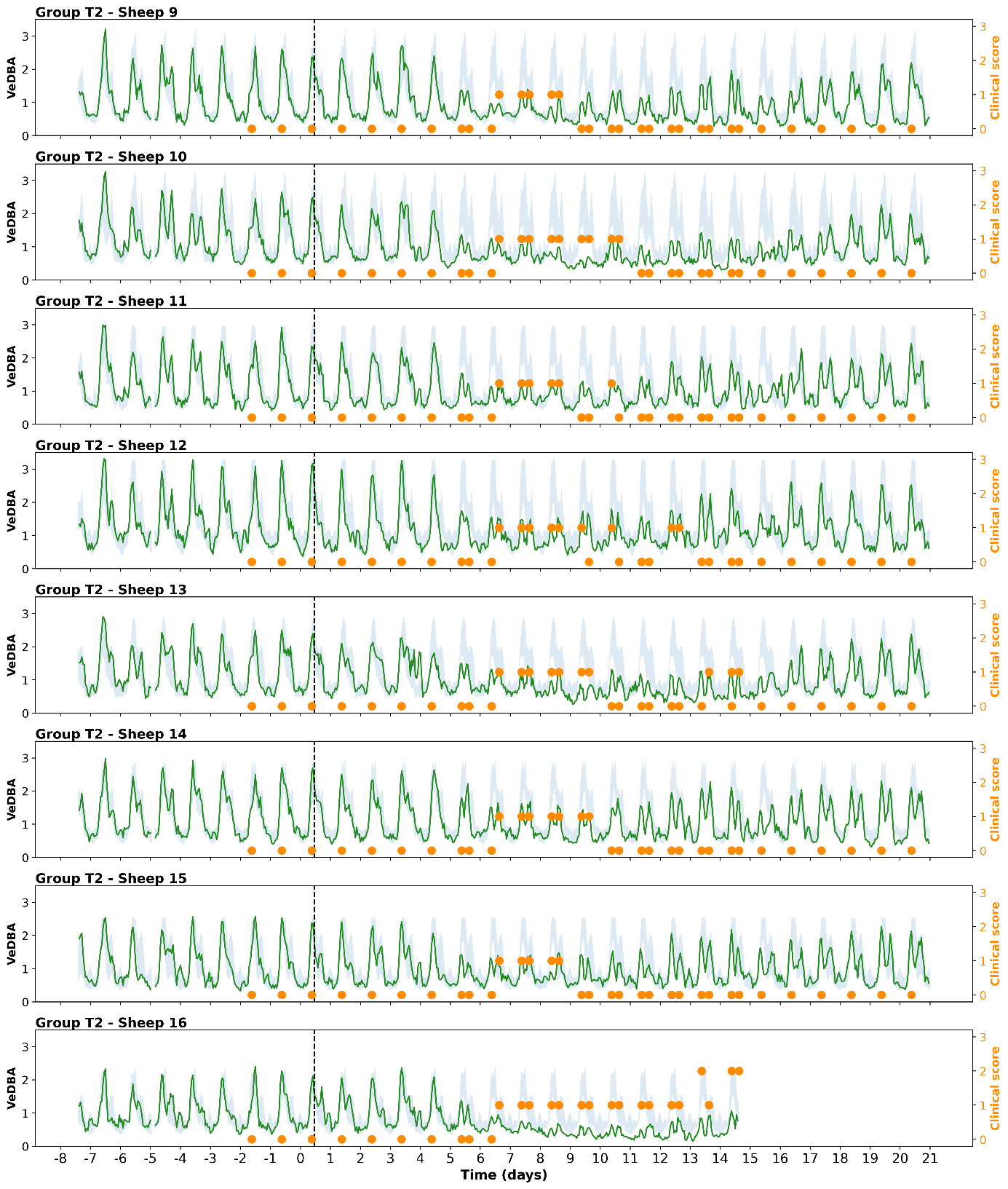
**

**Supplementary Figure S3.** (continued)

**
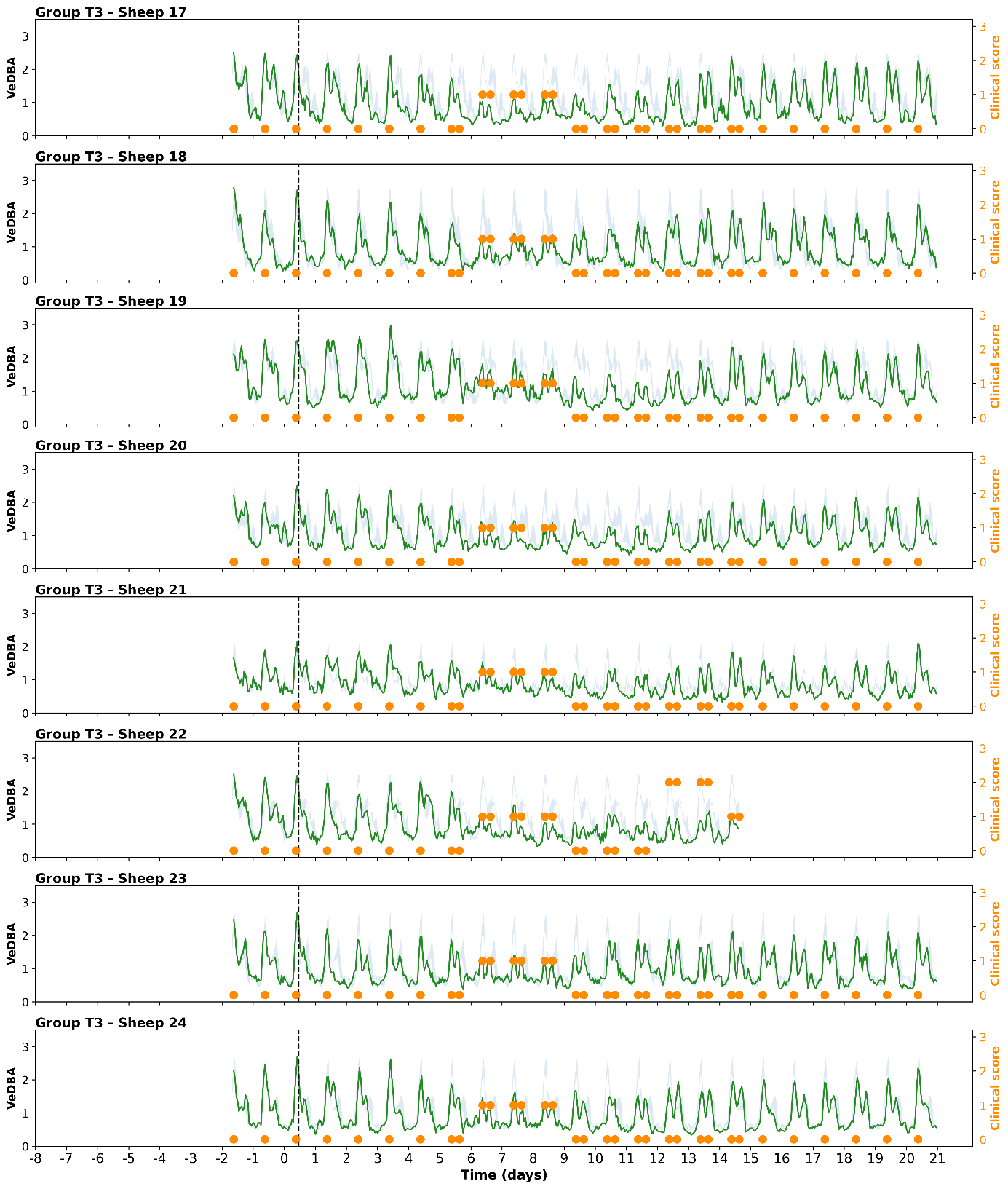
**

**Supplementary Figure S3.** (continued)
