# Supplementary material for: RAMSMART: a low-invasive system for real-time automated multi-species monitoring of livestock activity in research trials
Source: Front Vet Sci. 2026 Jun 22;13:1830138. doi: 10.3389/fvets.2026.1830138 (PMC13333429; doi:10.3389/fvets.2026.1830138)
Supplement: Supplementary file 4 [file Table_1.docx]

**Supplementary Table S1.** Battery life for different combinations of updating interval, broadcasting frequency and sampling frequency, showing the mean and standard deviation of three replicates for each combination.

| Updating interval (seconds) | Broadcasting frequency (Hz) | Sampling frequency (Hz) | Mean battery life (days) | SD battery life (days) |
| --- | --- | --- | --- | --- |
| 1 | 4 | 13 | 15.90 | 1.00 |
| 2 | 4 | 13 | 28.05 | 1.47 |
| 10 | 4 | 13 | 39.75 | 1.69 |
| 60 | 4 | 13 | 47.68 | 1.85 |
| 1 | 4 | 13 | 15.90 | 1.00 |
| 1 | 8 | 13 | 14.45 | 0.62 |
| 1 | 12 | 13 | 14.56 | 0.77 |
| 1 | 16 | 13 | 10.87 | 1.01 |
| 1 | 16 | 26 | 12.39 | 0.85 |
| 1 | 16 | 52 | 9.96 | 0.58 |
| 1 | 16 | 104 | 11.40 | 0.16 |
| 1 | 16 | 208 | 8.75 | 0.75 |
