# Supplementary material for: RAMSMART: a low-invasive system for real-time automated multi-species monitoring of livestock activity in research trials
Source: Front Vet Sci. 2026 Jun 22;13:1830138. doi: 10.3389/fvets.2026.1830138 (PMC13333429; doi:10.3389/fvets.2026.1830138)
Supplement: Supplementary file 5 [file Table_2.docx]

**Supplementary Table S2.** Data completeness and variation across sensors for test scenarios with eight sensors that were used simultaneously for twelve hours, with a varying updating interval (Upd. Interv.) and a varying broadcasting frequency (Broad. freq.).

|  |  |  | N packets received per hour | |  | Total N packets across all 12 hours | | |
| --- | --- | --- | --- | --- | --- | --- | --- | --- |
| Upd. Interv. (s) | Broad. freq. (Hz) | Sensor | Mean | SD |  | Expected | Missing | % missing |
| 60 | 1 | A | 55.8 | 0.6 |  | 674 | 5 | 0.7 |
| 60 | 1 | B | 56.6 | 0.9 |  | 684 | 5 | 0.7 |
| 60 | 1 | C | 57.2 | 0.8 |  | 690 | 3 | 0.4 |
| 60 | 1 | D | 57.3 | 0.5 |  | 693 | 5 | 0.7 |
| 60 | 1 | E | 58.2 | 1.1 |  | 704 | 5 | 0.7 |
| 60 | 1 | F | 59.8 | 0.8 |  | 721 | 3 | 0.4 |
| 60 | 1 | G | 60.1 | 0.7 |  | 726 | 5 | 0.7 |
| 60 | 1 | H | 62.2 | 1.4 |  | 758 | 11 | 1.5 |
| 60 | 4 | A | 56.2 | 0.4 |  | 674 | 0 | 0 |
| 60 | 4 | B | 57.1 | 0.3 |  | 685 | 0 | 0 |
| 60 | 4 | C | 57.6 | 0.5 |  | 691 | 0 | 0 |
| 60 | 4 | D | 57.8 | 0.5 |  | 693 | 0 | 0 |
| 60 | 4 | E | 58.6 | 0.5 |  | 703 | 0 | 0 |
| 60 | 4 | F | 60.1 | 0.3 |  | 721 | 0 | 0 |
| 60 | 4 | G | 60.6 | 0.5 |  | 727 | 0 | 0 |
| 60 | 4 | H | 63.2 | 0.5 |  | 759 | 0 | 0 |
| 60 | 8 | A | 56.2 | 0.4 |  | 674 | 0 | 0 |
| 60 | 8 | B | 57 | 0 |  | 684 | 0 | 0 |
| 60 | 8 | C | 57.6 | 0.5 |  | 691 | 0 | 0 |
| 60 | 8 | D | 57.8 | 0.5 |  | 693 | 0 | 0 |
| 60 | 8 | E | 58.6 | 0.5 |  | 703 | 0 | 0 |
| 60 | 8 | F | 60.2 | 0.4 |  | 722 | 0 | 0 |
| 60 | 8 | G | 60.6 | 0.5 |  | 727 | 0 | 0 |
| 60 | 8 | H | 63.2 | 0.5 |  | 759 | 0 | 0 |
| 60 | 12 | A | 56.2 | 0.4 |  | 674 | 0 | 0 |
| 60 | 12 | B | 57 | 0 |  | 684 | 0 | 0 |
| 60 | 12 | C | 57.5 | 0.5 |  | 690 | 0 | 0 |
| 60 | 12 | D | 57.8 | 0.5 |  | 693 | 0 | 0 |
| 60 | 12 | E | 58.6 | 0.5 |  | 703 | 0 | 0 |
| 60 | 12 | F | 60.2 | 0.4 |  | 722 | 0 | 0 |
| 60 | 12 | G | 60.5 | 0.5 |  | 726 | 0 | 0 |
| 60 | 12 | H | 63.2 | 0.5 |  | 759 | 0 | 0 |
| 60 | 16 | A | 56.2 | 0.4 |  | 674 | 0 | 0 |
| 60 | 16 | B | 57 | 0 |  | 684 | 0 | 0 |
| 60 | 16 | C | 57.6 | 0.5 |  | 691 | 0 | 0 |
| 60 | 16 | D | 57.7 | 0.5 |  | 692 | 0 | 0 |
| 60 | 16 | E | 58.6 | 0.5 |  | 703 | 0 | 0 |
| 60 | 16 | F | 60.2 | 0.4 |  | 722 | 0 | 0 |
| 60 | 16 | G | 60.5 | 0.5 |  | 726 | 0 | 0 |

**Supplementary Table S2.** (continued)

|  |  |  | N packets received per hour | |  | Total N packets across all 12 hours | | |
| --- | --- | --- | --- | --- | --- | --- | --- | --- |
| Upd. Interv. (s) | Broad. freq. (Hz) | Sensor | Mean | SD |  | Expected | Missing | % missing |
| 1 | 1 | A | 521.8 | 24.1 |  | 40256 | 33995 | 84.4 |
| 1 | 1 | B | 545.8 | 23.2 |  | 40847 | 34297 | 84 |
| 1 | 1 | C | 530.4 | 26.5 |  | 41148 | 34783 | 84.5 |
| 1 | 1 | D | 531.1 | 14.9 |  | 41297 | 34924 | 84.6 |
| 1 | 1 | E | 529.1 | 27.3 |  | 42001 | 35652 | 84.9 |
| 1 | 1 | F | 536.7 | 29.7 |  | 42924 | 36484 | 85 |
| 1 | 1 | G | 527.3 | 28.5 |  | 43418 | 37090 | 85.4 |
| 1 | 1 | H | 535.2 | 24.7 |  | 45298 | 38876 | 85.8 |
| 1 | 4 | A | 1792.2 | 35.8 |  | 40436 | 18929 | 46.8 |
| 1 | 4 | C | 1788.7 | 22.2 |  | 41432 | 19968 | 48.2 |
| 1 | 4 | D | 1788.4 | 35.4 |  | 41509 | 20048 | 48.3 |
| 1 | 4 | E | 1776.6 | 44.2 |  | 42198 | 20879 | 49.5 |
| 1 | 4 | F | 1814.2 | 28.8 |  | 43282 | 21511 | 49.7 |
| 1 | 4 | G | 1803.2 | 22.4 |  | 43569 | 21930 | 50.3 |
| 1 | 4 | H | 1815.8 | 29.5 |  | 45559 | 23770 | 52.2 |
| 1 | 8 | A | 2645.8 | 27.4 |  | 40437 | 8687 | 21.5 |
| 1 | 8 | B | 2673.4 | 37.2 |  | 41091 | 9010 | 21.9 |
| 1 | 8 | C | 2684 | 30 |  | 41436 | 9228 | 22.3 |
| 1 | 8 | D | 2667.4 | 26.1 |  | 41516 | 9507 | 22.9 |
| 1 | 8 | E | 2678.2 | 15.1 |  | 42204 | 10065 | 23.8 |
| 1 | 8 | F | 2708.3 | 18.5 |  | 43280 | 10780 | 24.9 |
| 1 | 8 | G | 2681.1 | 23.3 |  | 43588 | 11415 | 26.2 |
| 1 | 8 | H | 2717.6 | 18.4 |  | 45553 | 12942 | 28.4 |
| 1 | 12 | A | 3088.2 | 15.6 |  | 40444 | 3385 | 8.4 |
| 1 | 12 | B | 3129.2 | 19.6 |  | 41085 | 3534 | 8.6 |
| 1 | 12 | C | 3136.8 | 16.1 |  | 41442 | 3801 | 9.2 |
| 1 | 12 | D | 3126.2 | 22 |  | 41526 | 4011 | 9.7 |
| 1 | 12 | E | 3175 | 21.1 |  | 42212 | 4112 | 9.7 |
| 1 | 12 | F | 3230 | 22 |  | 43287 | 4527 | 10.5 |
| 1 | 12 | G | 3259.7 | 28.7 |  | 43595 | 4479 | 10.3 |
| 1 | 12 | H | 3337.8 | 25.4 |  | 45559 | 5506 | 12.1 |
| 1 | 16 | A | 3267.7 | 12.9 |  | 40435 | 1223 | 3 |
| 1 | 16 | B | 3318.2 | 9.7 |  | 41072 | 1254 | 3.1 |
| 1 | 16 | C | 3341.2 | 13.4 |  | 41442 | 1347 | 3.3 |
| 1 | 16 | D | 3359.2 | 9.8 |  | 41561 | 1251 | 3 |
| 1 | 16 | E | 3407 | 8.7 |  | 42218 | 1334 | 3.2 |
| 1 | 16 | F | 3477.8 | 8.8 |  | 43300 | 1567 | 3.6 |
| 1 | 16 | G | 3496.1 | 10.2 |  | 43581 | 1628 | 3.7 |
| 1 | 16 | H | 3384.6 | 20 |  | 45567 | 4952 | 10.9 |
