# Supplementary material for: RAMSMART: a low-invasive system for real-time automated multi-species monitoring of livestock activity in research trials
Source: Front Vet Sci. 2026 Jun 22;13:1830138. doi: 10.3389/fvets.2026.1830138 (PMC13333429; doi:10.3389/fvets.2026.1830138)
Supplement: Supplementary file 6 [file Table_3.docx]

**Supplementary Table S3.** Data completeness across animals for the three (pig, calf, and sheep) trials.

|  |  |  |  | Total N packets across monitoring period | | |
| --- | --- | --- | --- | --- | --- | --- |
| Trial | Animal | Treatment | Pen | Expected | Missing | % missing |
| Pig | 1 | T1 | Room1 | 18678 | 752 | 4.03 |
| Pig | 2 | T1 | Room1 | 19848 | 1030 | 5.19 |
| Pig | 3 | T1 | Room1 | 18174 | 282 | 1.55 |
| Pig | 4 | T1 | Room1 | 18965 | 739 | 3.9 |
| Pig | 5 | T1 | Room1 | 19664 | 1137 | 5.78 |
| Pig | 6 | T1 | Room1 | 19029 | 795 | 4.18 |
| Pig | 7 | T2 | Room2 | 20422 | 399 | 1.95 |
| Pig | 8 | T2 | Room2 | 32898 | 357 | 1.09 |
| Pig | 9 | T2 | Room2 | 31792 | 403 | 1.27 |
| Pig | 10 | T2 | Room2 | 32321 | 409 | 1.27 |
| Pig | 11 | T2 | Room2 | 32320 | 214 | 0.66 |
| Pig | 12 | T2 | Room2 | 31236 | 184 | 0.59 |
| Pig | 13 | T3 | Room3 | 19359 | 498 | 2.57 |
| Pig | 14 | T3 | Room3 | 18842 | 474 | 2.52 |
| Pig | 15 | T3 | Room3 | 18393 | 301 | 1.64 |
| Pig | 16 | T3 | Room3 | 19035 | 222 | 1.17 |
| Calf | 1 | T1 | Pen1 | 36758 | 240 | 0.65 |
| Calf | 2 | T1 | Pen1 | 37686 | 182 | 0.48 |
| Calf | 9 | T2 | Pen1 | 39501 | 282 | 0.71 |
| Calf | 10 | T2 | Pen1 | 35912 | 184 | 0.51 |
| Calf | 17 | T3 | Pen1 | 37419 | 332 | 0.89 |
| Calf | 18 | T3 | Pen1 | 40007 | 361 | 0.9 |
| Calf | 5 | T1 | Pen2 | 39799 | 403 | 1.01 |
| Calf | 6 | T1 | Pen2 | 37663 | 528 | 1.4 |
| Calf | 13 | T2 | Pen2 | 38338 | 603 | 1.57 |
| Calf | 14 | T2 | Pen2 | 38322 | 782 | 2.04 |
| Calf | 21 | T3 | Pen2 | 37754 | 900 | 2.38 |
| Calf | 22 | T3 | Pen2 | 37634 | 879 | 2.34 |
| Calf | 3 | T1 | Pen3 | 37020 | 562 | 1.52 |
| Calf | 4 | T1 | Pen3 | 37296 | 833 | 2.23 |
| Calf | 11 | T2 | Pen3 | 36390 | 587 | 1.61 |
| Calf | 12 | T2 | Pen3 | 22371 | 582 | 2.6 |
| Calf | 19 | T3 | Pen3 | 36976 | 833 | 2.25 |
| Calf | 20 | T3 | Pen3 | 37738 | 1014 | 2.69 |
| Calf | 7 | T1 | Pen4 | 38828 | 1618 | 4.17 |
| Calf | 8 | T1 | Pen4 | 38451 | 1498 | 3.9 |
| Calf | 15 | T2 | Pen4 | 36858 | 727 | 1.97 |
| Calf | 16 | T2 | Pen4 | 35979 | 1110 | 3.09 |
| Calf | 23 | T3 | Pen4 | 40329 | 1633 | 4.05 |
| Calf | 24 | T3 | Pen4 | 38579 | 1123 | 2.91 |

**Supplementary Table S3.** (continued)

|  |  |  |  | Total N packets across monitoring period | | |
| --- | --- | --- | --- | --- | --- | --- |
| Trial | Animal | Treatment | Pen | Expected | Missing | % missing |
| Sheep | 1 | T1 | Room1 | 25086 | 0 | 0 |
| Sheep | 2 | T1 | Room1 | 23335 | 3 | 0.01 |
| Sheep | 3 | T1 | Room1 | 21177 | 18 | 0.08 |
| Sheep | 4 | T1 | Room1 | 18904 | 0 | 0 |
| Sheep | 5 | T1 | Room1 | 24926 | 5 | 0.02 |
| Sheep | 6 | T1 | Room1 | 26929 | 0 | 0 |
| Sheep | 7 | T1 | Room1 | 26321 | 0 | 0 |
| Sheep | 8 | T1 | Room1 | 26527 | 1 | 0 |
| Sheep | 9 | T2 | Room2 | 38639 | 0 | 0 |
| Sheep | 10 | T2 | Room2 | 39903 | 0 | 0 |
| Sheep | 11 | T2 | Room2 | 39240 | 0 | 0 |
| Sheep | 12 | T2 | Room2 | 40067 | 1 | 0 |
| Sheep | 13 | T2 | Room2 | 42124 | 0 | 0 |
| Sheep | 14 | T2 | Room2 | 40104 | 0 | 0 |
| Sheep | 15 | T2 | Room2 | 40656 | 0 | 0 |
| Sheep | 16 | T2 | Room2 | 31539 | 73 | 0.23 |
| Sheep | 17 | T3 | Room3 | 31911 | 0 | 0 |
| Sheep | 18 | T3 | Room3 | 31901 | 0 | 0 |
| Sheep | 19 | T3 | Room3 | 32954 | 0 | 0 |
| Sheep | 20 | T3 | Room3 | 32678 | 0 | 0 |
| Sheep | 21 | T3 | Room3 | 31386 | 0 | 0 |
| Sheep | 22 | T3 | Room3 | 21981 | 17 | 0.08 |
| Sheep | 23 | T3 | Room3 | 34438 | 0 | 0 |
| Sheep | 24 | T3 | Room3 | 32656 | 0 | 0 |
